# A genome-wide association study identified SNP markers and candidate genes associated with morphometric fruit quality traits in mangoes

**DOI:** 10.1186/s12864-025-11278-6

**Published:** 2025-02-07

**Authors:** Shamseldeen Eltaher, Jin Li, Barbie Freeman, Sukhwinder Singh, Gul Shad Ali

**Affiliations:** 1 Subtropical Horticulture Research Station (SHRS), United States Department of Agriculture, Agricultural Research Service (USDA-ARS), Miami, FL USA; 2 Department of Plant Biotechnology, Genetic Engineering and Biotechnology Research Institute (GEBRI), University of Sadat City (USC), Sadat City, 32897 Egypt

**Keywords:** Mango germplasm, Marker traits association, Population structure, Single nucleotide polymorphism, Fruit development

## Abstract

**Background:**

Mangoes (*Mangifera indica* L.) are a widely grown fruit tree crop across the world, but breeding new varieties can take 15-20 years due to its long juvenile period and high heterozygosity. Marker-assisted selection can accelerate breeding new mango cultivars with desirable traits for fruit quality, storage, horticulture, pest and disease resistance, and nutrition.

**Results:**

To achieve this, a genome-wide association study (GWAS) was conducted to discover molecular markers for 14 morphometric and economically important fruit traits of 161 mango accessions with diverse genetic backgrounds. These traits included pulp and brix; fruit weight, length, thickness, and width; stone weight, length, thickness, and width; and seed weight, length, thickness, and width. In this report, we employed the fixed and random model circulating probability unification (FarmCPU) model for conducting GWAS using 135,079 high-quality SNP markers. These analyses revealed 103 SNPs that were significantly associated with these traits. Of these markers, 7 were commonly associated with different traits, while 96 markers were uniquely associated with specific traits.

**Conclusions:**

To choose the most promising mango accessions for future breeding and for closing genetic gaps among the accessions and increasing genetic diversity, a new selection method is suggested based on phenotypic traits such as high-yielding mango fruit cultivars, number of reference alleles, and genetic distance among the selected genotypes. Based on these criteria, 20 accessions were identified as the most promising parents for crossing to produce high mango yield. Gene annotation of the significant markers revealed candidate genes coding for important proteins, enzymes, and transcription factors associated with fruit development traits.

**Supplementary Information:**

The online version contains supplementary material available at 10.1186/s12864-025-11278-6.

## Background

Mangoes (*Mangifera indica* L.) are among the most widely consumed fruits worldwide and are referred to as the "king of fruits" [1, 2]. Mangoes are grown in more than 100 countries in tropical and subtropical areas around the world from latitudes 37° N in Sicily to 33° S in South Africa [3]. India, Indonesia, China, Pakistan, and Mexico are the top five producing countries [1]. Although production data only on mangoes are not reported, when combined with mangosteens, and guavas, world production of mangos, reached slightly over 59 million metric tons in 2022, an increase from approximately 57 million tons in 2021 ( https://www.fao.org/faostat/). Mangoes rank as the fifth most produced fruit crop worldwide. Mangoes are members of the *Anacardiaceae* family and genus *Mangifera* [4]. Although *M. indica* is the most commonly planted species, there are numerous other *Mangifera* species that also produce edible fruits, including *M. altissima* (also known as Paho) and *M. rubropetela* (also known as Red Petaled Mango) [1, 5]. Mango fruits has a pleasant aroma and are mostly eaten fresh, however, a substantial amount is also process into making powder, juice, jam, and nectar [2, 6].

Various fruit quality factors such as weight, size, colour, aroma, flavour, taste, and texture influence consumer’s preference and determine commercial value of mango cultivars. However, in response to increasing consumer interest in health-promoting foods, nutritional content has emerged as an additional factor in consumer preference [7]. Furthermore, the quality of mangoes and their bioactive compounds are affected by the ripening stage during harvest and during storage [7, 8]. Older *M. indica* varieties and other species that thrive in North-East India, the Andaman Islands, and across South-East Asia demonstrate that the fruit of early domesticated mango types is often small [9]. Mango fruit size has long been a top breeding goal, and as a result, highly traded mango types now average 400 g in size [5, 9–13]. Transcription factors and plant hormones affect fruit growth in both climacteric and non-climacteric fruits. Different genes regulate fruit size, shape, and weight, and abnormal expression of these genes can cause physiological disorders [14]. Mango fruits are characterized as climacteric, in which ripening continues after harvest primarily attributed to an increased production of autocatalytic ethylene and enhanced respiration. Molecular markers such as simple-sequence-repeat (SSRs) associated with fruit weight, width, volume, total soluble solids (TSS), titratable acidity, ascorbic acid, and total sugars, together with decreasing sugars, are useful in the selection procedure for *M. indica* varieties and seedlings that exhibit desirable fruit traits [15, 16].

Due to the long juvenile period and cost-prohibitive horticultural operations involved with generating, maintaining and evaluating fruit quality traits in mango breeding populations, development and release of new mango cultivars is very slow. In addition, the polygenic nature of most fruit quality traits further exacerbates selection of these traits. Development and application of high-density molecular markers for fruit quality traits at the seedling stage can significantly accelerate and simplify breeding new mango cultivars. Towards the application of marker-assisted selection (MAS) and quantitative traits loci (QTLs) mapping, several mango genetic maps based on restriction fragment length polymorphism (RFLP) and amplified fragment length polymorphism (AFLP) have been created [17–19]. Using 80K SNP chip, a mango linkage map as well as QTL mapping for fruit color and firmness have been reported, which, in addition to the work reported in this manuscript, provides foundations for future studies [20]. However, the small number of mapping populations and markers, does not allow drawing valid conclusions and application of markers in breeding programs. Recently, genome sequencing and using whole genome resequencing of more than a total of 250 different cultivars [1, 21–23], high density single nucleotide polymorphic (SNP) markers have been developed, which has started to discover markers associated with fruit quality traits with high confidence using genome-wide association studies (GWAS) [21]. For example, these markers are expected to be developed into cost-effective platforms such as competitive allele specific PCR (KASP) and applied in marker assisted selection in breeding programs. Among these, those markers associated with qualitatively inherited traits that display mendelian segregation such as polyembryony and disease resistance *R* genes are likely to be used more widely followed by those associated with quantitative traits that display polygenic segregation using genomic selection (GS) tools.

Different genes are involved in fruit development traits such as Mitogen-activated protein kinases (MAPKs) [24]. Serine/threonine protein kinases called MAPKs are involved in the phosphorylation-based upstream and downstream control of signalling cascades. Plant fruit growth is mediated by MAPK cascades through the ethylene signalling system [14, 24, 25]. Ten MAPKK and 77 MAPKKK genes were shown to be involved in tissue growth, fruit development and ripening, and response to abiotic stressors such as cold, drought, and salt, according to an accurate transcriptome of bananas [26]. Dillon et al., [22] identified two genomic regions, one on linkage group four (LG4) and one on LG7 containing 28 candidate genes associated with fruit size in the mango mapping population ‘Tommy Atkins’ x ‘Kensington Pride’ during complete final ‘Tommy Atkins’ genome assembly.

GWAS is a powerful tool for identifying markers associated with complex traits in unstructured populations, such as breeding lines and germplasm collections [27–29]. Compared to bi-parental populations, these GWAS panels display higher recombination rates thus significantly improving mapping resolutions [22, 23]. Compared to annual crops, due to the complexity of fruit crop genomics, high heterozygosity, long generation times, and limited genomics resources, identifying associated genetic markers in fruit crops is challenging [24, 25]. The application of GWAS and marker-assisted selection (MAS) tools to fruit quality traits that in fruit tree crops can be measured only after several years of vegetative growth, have been limited [30–33]. In this study, using whole genome resequencing, we identified high-quality high-density SNP markers in a genetically diverse panel of *M. indica* accessions. Utilizing these markers, we identified markers associated with morphometric fruit-quality traits that will be useful for marker-assisted selection in mango breeding programs.

## Materials and methods

### Plant material

The cultivars used in this study are maintained at the United States Department of Agriculture, Agricultural Research Service (USDA-ARS), Subtropical Horticulture Research Station (SHRS) located in Miami, Florida. The average temperature was 75.60°F, ranging from 62°F to 90°F, with an average annual precipitation of 60.51 inches (Miami-Dade County Weather). A total of 269 *M. indica* accessions originating from various parts of the world are maintained at this station. Of these, complete molecular markers data and substantially diverse phenotypic data for a set of 161 accessions were available, which were used in this study (see below).

### Mango morphometric fruit quality traits

The selected 161 accessions displayed extensive variation in morphological traits. Nineteen accessions produced small fruit (< 200g), 115 accessions produced medium size fruit (200-500g), and 27 accessions produced large fruits (> 500g). A comprehensive list of all *Mangifera sp*. accessions included in this study is provided in Table S1. Mature mango fruits (12–36) were collected over a nine-year period (2013–2022), and 14 morphometric fruit quality traits were evaluated. These traits included fruit weight, fruit length, fruit thickness, fruit width, stone weight, stone length, stone thickness, stone width, seed weight, seed length, seed thickness, seed width, pulp, and Brix, which are commercially important and influence consumer’s preference and marketability. The pulp, which denotes the mango fruit's soft, juicy, or fleshy part, was estimated as the quotient between fruit weight and stone weight. Conversely, brix is a measure of the dissolved solids in a liquid and is commonly employed to determine the dissolved sugar content of an aqueous solution. One degree Brix equals 1 gram of sucrose in 100 grams of the solution. Brix measurements were obtained using a PAL-1 refractometer (ATAGO, WA, USA). The International Plant Genetic Resources Institute (IPGRI) measurement guidelines were followed for the traits reported in this study [34]. The Shapiro-Wilk test was conducted to identify the normal distribution for each trait and the histograms were visualized using R software. The Pearson’s correlation coefficient was calculated among all traits using the “cormat” code in R software and the upper triangle heatmap was visualized using R “ggplot2” package.

###  DNA extraction and SNP calling

Biosearch Technologies, LGC (Middleton, WI) used a unique procedure to isolate DNA from leaf samples of all mango accessions. Using a Quant-iTTM dsDNA Assay Kit, the quality of the DNA was evaluated. To prepare the library, 500 ng of gDNA were shared using a Covaris® LE220 Focused Ultrasonicator. Then, using the Biosearch Technologies NxSeq® UltraLow and NxSeq HT Dual Indexing Kit, normalization was performed for the creation of shotgun fragment libraries. Libraries were sequenced on an Illumina NovaSeq™ 6000 instrument at the University of Wisconsin Biotechnology Center utilizing the Illumina 150x PE platform. The reads underwent simultaneous quality trimming and adapter clipping with a minimal final length of 50 bp and a mean Q score of 20, utilizing a 10 bp sliding window. Using BWA-mem with default parameters, cleaned reads were aligned to the ‘Alphonso’ reference genome CATAS_Mindica_2.1 (GCA_011075055.1) [1]. Duplicates with default parameter settings were marked with the Genome Analysis Toolkit (GATK) Picard Tools [35].

SNPs were called for each sample independently using DeepVariant v1.4.0 [36] and --model type = WGS using a Singularity container that was constructed from a pre-built docker container (https://github.com/google/deepvariant). Using GLnexus v1.2.7. with --config DeepVariantWGS for Whole Genome Sequencing, the resultant gVCF files were merged into a single vcf file. GLnexus was run on a Singularity container, which was constructed from a docker container (https://github.com/dnanexus-rnd/GLnexus). Using vcftools, SNPs were filtered with the following criteria: quality ≥ 30, missing call rate = 0.9, min_depth = 6, max_depth = 1000, minor allele frequency = 0.05. A set of 135,079 high-quality SNPs (MAF > 0.05, missed call rate < 0.1, median gap = 0.306 kb, average gap = 2.645 kb) was obtained. The SNP distribution density on chromosomes was visualized using CMplot [37] and custom R code based on the Alphonso reference genome (GCA_011075055.1) CATAS_Mindica_2.1 [1]. The high quality 135,079 SNPs markers are persented in Table S2.

### Analysis of linkage disequilibrium (LD) and population structure

The analysis of linkage disequilibrium (*r*^*2*^) of each pair of the 135.079 SNPs was calculated using TASSEL v.5.2.5 software [38]. Moreover, for each chromosome, the LD was also calculated to understand the structure of LD in the current population. The significant LD between each marker pair was determined using a Bonferroni correction set at a significance level at 0.01. The LD decay was calculated according to Remington et al. [39] using R version 4.3.3.

A Bayesian model-based method using 135,079 SNPs was employed to assess the potential number of subpopulations in the accessions reported in this study. The population structure study was conducted using STRUCTURE 3.4.0, as described [40] using k values ranging from 1 to 10. For each k-value, three independent analyses were conducted with a burn-in iteration of 100,000, followed by 100,000 Markov chain Monte Carlo (MCMC) replications [41]. The optimal value of k for the present population was calculated using STRUCTURE SELECTOR [42] (https://lmme.ac.cn/StructureSelector/). The fixation index (F_ST_) and the expected heterozygosity (H_e_), which were determined using STRUCTURE 3.4.0 [40], represent the proportion of genetic variation within a subpopulation compared to the total genetic variation.

### Genome-wide association studies (GWAS)

The GWAS were performed for all traits using the rMVP R package [43]. In this report, we used three different GWAS models: (1) the Fixed and random model Circulation Probability Unification (FarmCPU), (2) the Generalized Linear Model (GLM), and (3) the Mixed Linear Model (MLM). The best fitted model for each trait were selected based on expected and observed *p*-values in QQ-plots. To account for structuring and kinship (Kin) in the data, principal component analysis (PCA), and PCA + Kin were added to each model separately. Combination of stepwise regression (fixed effect models) and mixed linear models enhances efficiency of FarmCPU. The FarmCPU model avoids the confusion between kinship and the genes driving an attribute in a mixed linear model by replacing kinship with a set of markers linked with the causal genes. According to X. Liu et al., [44], to prevent model overfitting, the related markers are optimized using a maximum likelihood technique in MLM with variance and covariance structures defined by the linked markers. Significant markers associated with the traits were discovered using a *p*-value < 0.0001. The targeted marker allele was identified as the one that increases the traits under investigation.

### Candidate genes identification and gene ontology enrichment analysis

Genes closely located to significant markers were identified and further examined for their molecular and biological function as follows. Using the GFT file extracted from https://www.ncbi.nlm.nih.gov/datasets/genome/GCF_011075055.1/ for the "Alphonso" reference genome (CATAS_Mindica_2.1;GCA_011075055.1), custom Python scripts were designed to verify the genomic position of each markers to specific genes [1]. TBtools [45] was used to perform the Kyoto Encyclopedia of Genes and Genomes (KEGG) pathway and Gene Ontology (GO) enrichment analysis. The GO ID and KEGG ID was extracted using eggNogmapper (http://eggnog-mapper.embl.de/). The signaling pathways were drawn using the KEGG web program (https://www.genome.jp/kegg/pathway.html).

## Results

### Phenotypic analysis of the mango varieties

The descriptive analyses including minimum, maximum, and mean for all genotypes are presented in Table 1. The distribution of 14 morphometric fruit traits data for all accessions is presented in Figure S1. Normal frequency distributions were observed in most of the morphometric traits with a little skewness for some traits using Shapiro-Wilk test (Table S1). Wide ranges were observed in fruit weight from 80.71 to 931.33 g, fruit length from 61.88 to 189.68 mm, fruit thickness from 48.41 to 111.25 mm, and fruit width from 38.33 to 97.04 mm. The stone weight and stone length also displayed a wide range of values, ranging from 13.44 to 57.46 g and 39.62 to 156.17 mm, respectively. For stone thickness, the range was 25.74 to 54.27 mm, while for stone width, it was 12.29 to 27.14 mm. Furthermore, there was a broad variation in the seed size attributes, which ranged from 6.63to 36.49 g for seed weight, 35.68 to 78.08 mm for seed length, 18.02 to 40.66 mm for seed thickness, and 10.15 to 23.52 mm for seed width. Furthermore, there was significant variance in the brix values across the mango cultivars, ranging from 9.92 to 22.40, and the pulp, ranging from 3.82 to 28.80.

**Table 1:** Descriptive statistics for morphometric fruit quality traits in Mango

Trait	Mean	Min.	Max.	SD	SE
**Fruit Weight (g)**	359.83	80.71	931.33	147.57	11.63
**Fruit Length (mm)**	105.13	61.88	189.68	21.23	1.67
**Fruit Width (mm)**	79.11	48.41	111.25	11.84	0.93
**Fruit Thickness (mm)**	71.15	38.33	97.04	11.05	0.87
**Stone Weight (g)**	31.43	13.44	57.46	8.56	0.67
**Stone Length (mm)**	82.07	39.62	156.17	19.87	1.61
**Stone Width (mm)**	38.72	25.74	54.27	5.38	0.42
**Stone Thickness (mm)**	19.48	12.29	27.14	2.48	0.20
**Seed Weight (g)**	18.74	6.63	36.49	5.41	0.43
**Seed Length (mm)**	56.34	35.68	78.08	8.19	0.65
**Seed Width (mm)**	29.95	18.02	40.66	3.91	0.31
**Seed Thickness (mm)**	15.83	10.15	23.52	2.14	0.17
**Pulp**	11.54	3.82	28.80	3.94	0.31
**Brix**	16.10	9.92	22.40	2.42	0.19

*Min *Minimum value observed, *Max *Maximum value observed, *SD *Stander deviation, *SE *Stander error of the mean

The Pearson’s correlation coefficient analysis among all morphometric fruit quality traits is presented in Figure S2. These analyses revealed varying but high and significant correlation among most traits except the brix and pulp. The highest strong positive correlation was found between fruit width and fruit thickness (*r* = 0.95***) followed by fruit length and stone length (*r* = 0.94***), then fruit weight and fruit width (*r* = 0.89***). No significant or very low significant correlations were observed between brix and the rest of the traits. A negative correlation was observed between fruit thickness and brix (*r*=−0.35**) and fruit width and brix (*r*=−0.33**)

### Linkage disequilibrium and population structure analysis

**Fig. 1:**
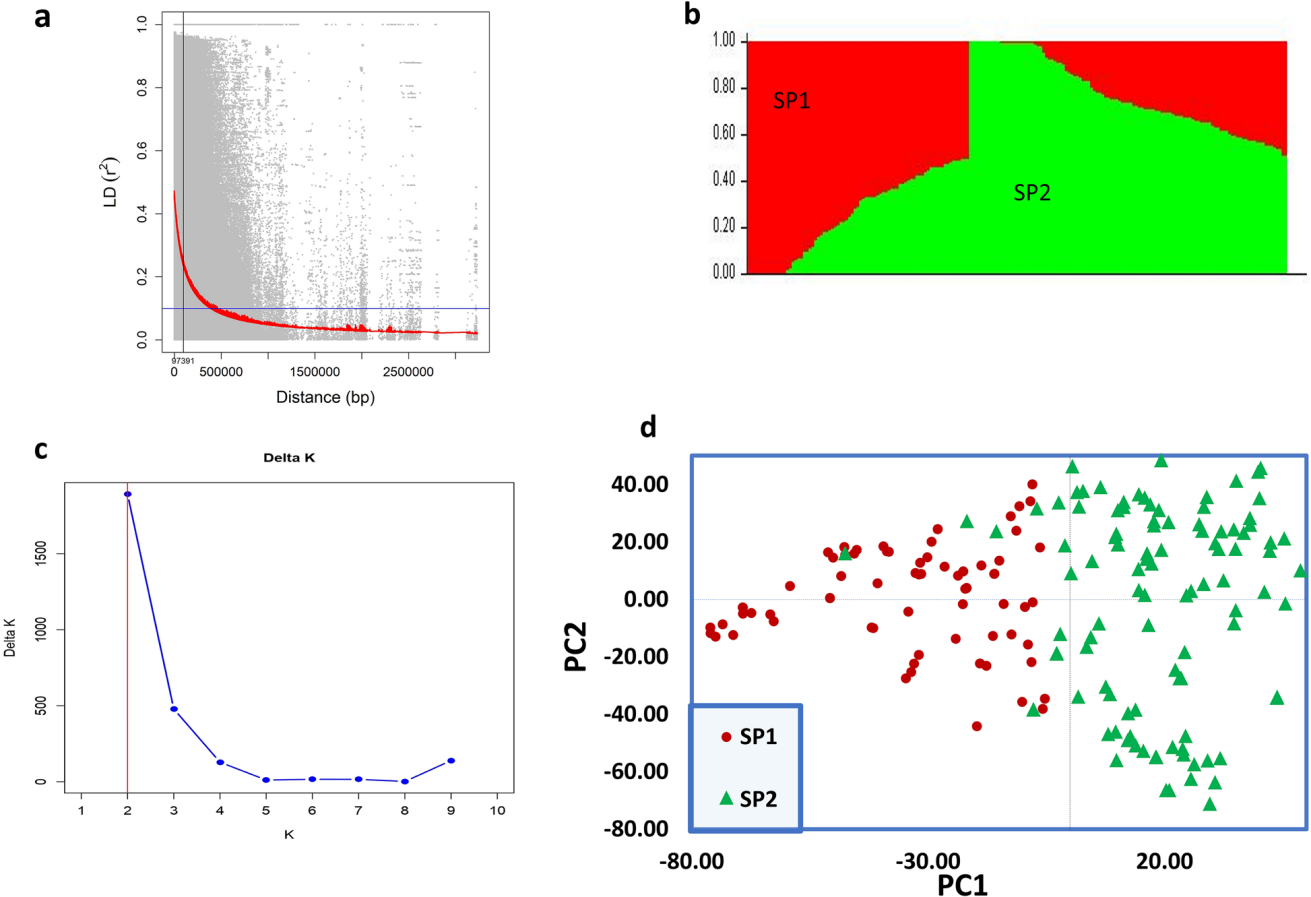
Genetic diversity and population structure analysis of mango cultivars. **a** Scatterplot showing linkage disequilibrrum (LD) decay estimated by plotting (r^2^) against genetic distance (bp) in 161 mango accessions. The green vertical line indicates the threshold point where LD dropped to 50% of its maximum value. The LD decay value at the cutoff point is indicated on the x-axis with green font. **b** The estimated population structure of 161 mango accessions using k=2; on (k=2) SP refers to subpopulation using STRUCTURE. **c** Delta (Δ)K for determining the optimal number of subpopulations (k=2). **d** PCA scatter plot based on the first two components

LD was estimated by calculating the squared correlation coefficient (r^2^) for all the 135,079 SNPs markers. Genome-wide LD decayed with increased genetic distance. Specifically, LD decayed to its half at 3.73 Mb (37391 bp) for whole genome (Fig. 1a). The model-based analysis of population structure along with the Delta K method of Evanno et al., [34] revealed the presence of two genetically distinct clusters, K = 2 Fig. 1b, which corresponded to the majority of cultivars from the United States, India, Southeast Asia and Caribbean countries. These results are illustrated in Fig. 1C, which shows a clear peak (Δ)K = 2. With the arbitrary cutoff value of 65% ancestry for assignment, 73 cultivars (45.34%) were attributed to one cluster (SP1) and 88 cultivars (54.65%) to the other cluster (SP2). The germplasm collection of mangoes in our study includes cultivars that were developed from India, Southeast Asia, South America and the Pacific islands, Florida, Egypt, and Israel. The PCA showed that the 161 mango accessions were divided into two different clusters (Fig. 1d). A cluster in red color contained most Indian accessions, as well as those from Southeast Asia, China, and Egypt (SP1), and the cluster in green contained accessions from the United States, South America, Israel, and the Caribbean (SP2). A significant genetic variance was detected between the two subpopulations, as well as the average genetic distance (expected heterozygosity H_e_) among mango genotypes within each subpopulation (Table 2). The maximum H_e_ was observed in SP2, with a value of 0.305, while SP1 exhibited a score of 0.281. The F_ST_ was computed to evaluate the population substructure and is recognized as the most effective measure for analyzing the overall genetic variance between the two subpopulations. The F_ST_ values for SP1 and SP2 were 0.247 and 0.256, respectively.

**Table 2:** Population structure analysis for genetic differentiation in 161 mango accessions

Sub Population	FST	Exp. Heterozygosity	No. of accession
SP1	0.247	0.281	79
SP2	0.256	0.305	113

*FST *Fixation Index (significant divergences), *Exp. Heterozygosity *Expected heterozygosity (average distances), *No. of accession *Number of genotypes in each subpopulation

### Genome wide association studies for morphometric fruit quality traits

**Fig. 2:**
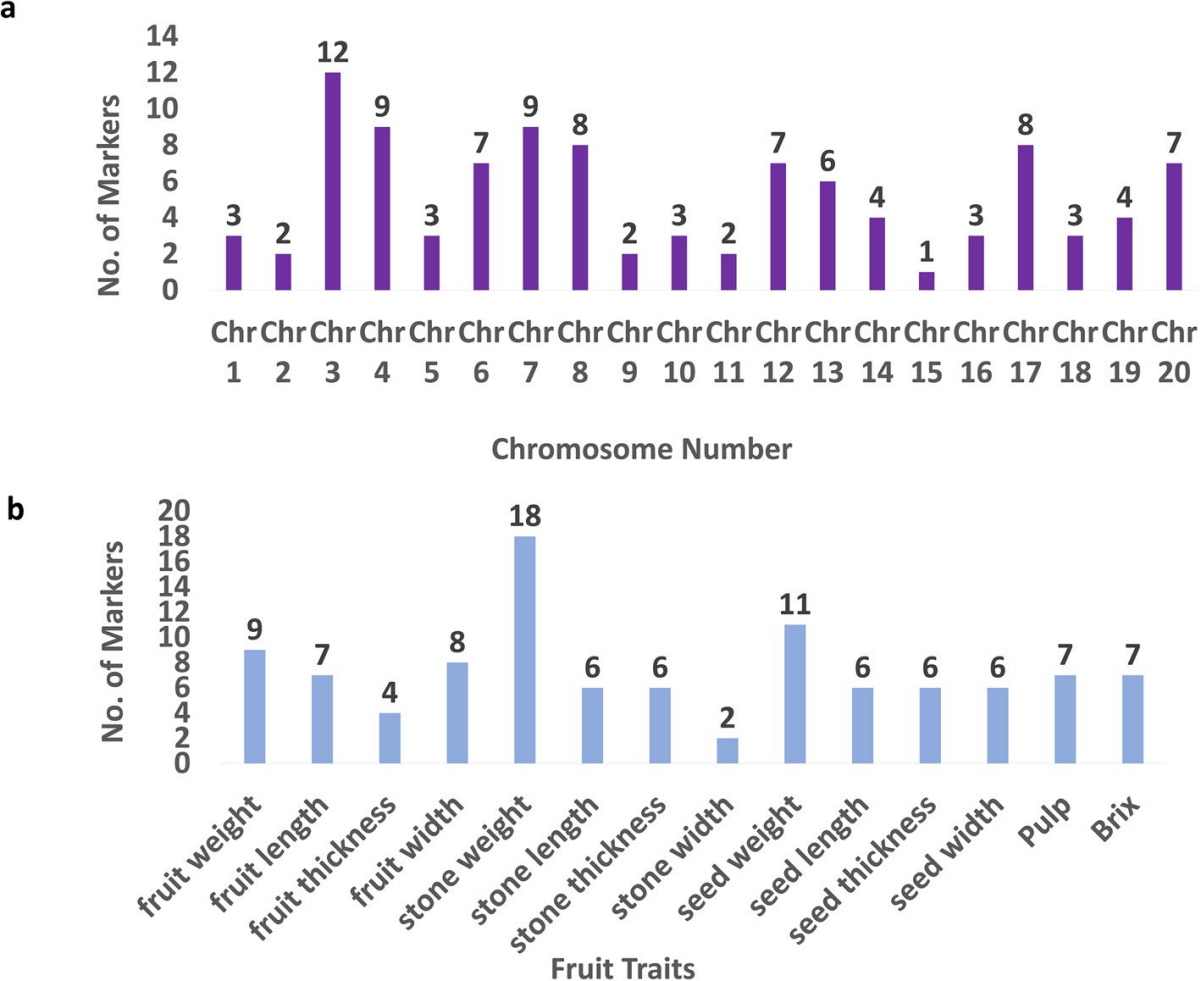
lot Distribution of alleles associated with mango fruit quality traits. **a** distribution of 103 significant SNPs across mango chromosomes **b** the distribution of 103 significant markers across the 14 morphometric fruit quality traits

**Fig. 3:**
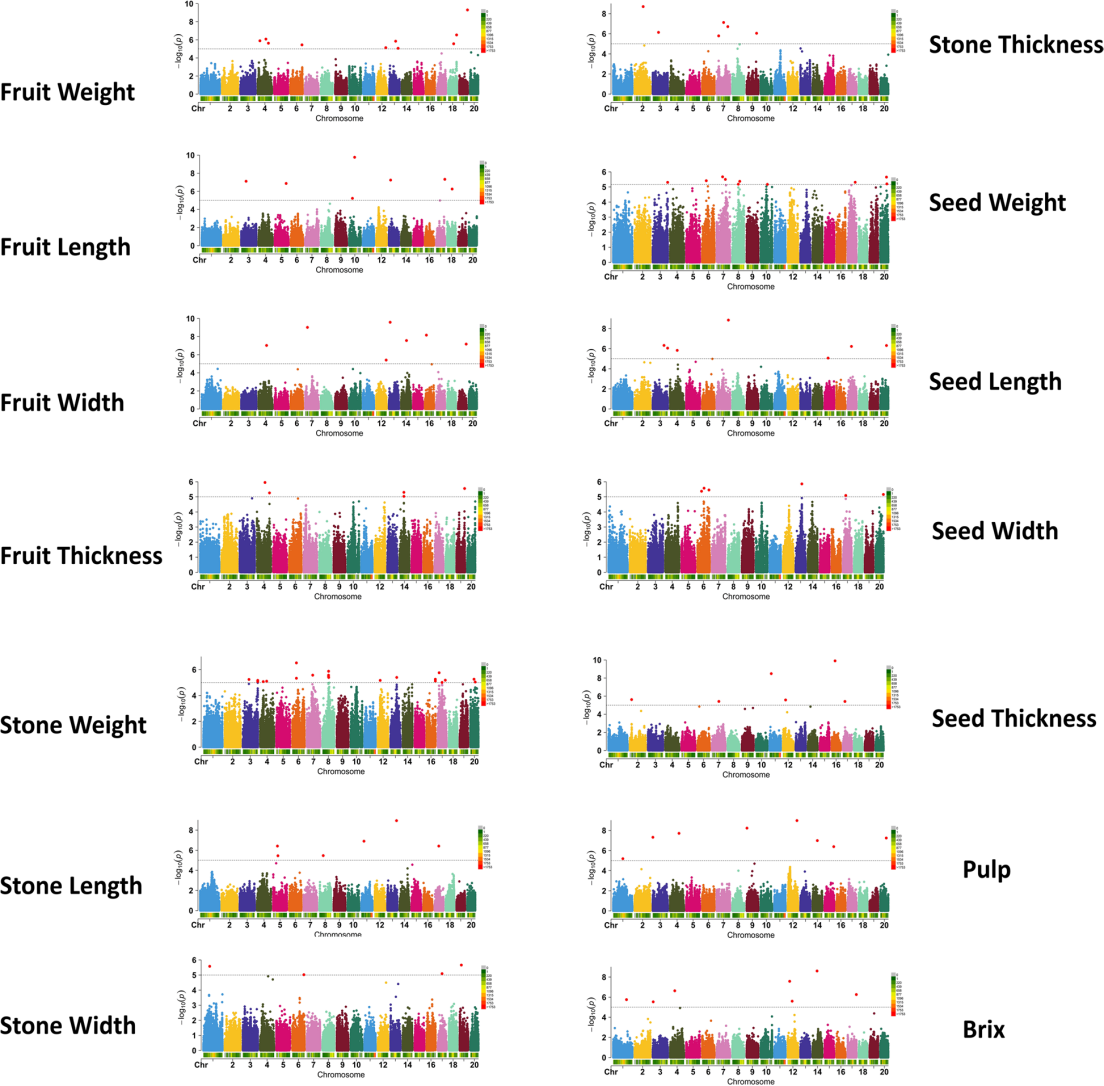
Manhattan plots displaying SNP marker-trait associationsed for 14 morphometric fruit traits using FarmCPU GWAS model. Analyses were derived from 135,079 SNPs markers distributed across 20 chromosomes. Significant SNPs surpassing the -log_10_(*p*) significance threshold values, indicated by horizontal lines, are highlighted in red. Color bars on the top right represent the distribution of SNP markers on each chromosome displayed at the bottom of each chromosome bar graph

The GWAS results for FarmCPU model showed 103 significant SNPs associated with 14 morphometric fruit quality traits. Even though the GLM model identified 260 significant markers, the comparison of expected and observed *p* values in the QQ plot analysis demonstrated that FarmCPU was the most effective GWAS model for each trait (Figure S3). On the other hand, the MLM model revealed just one significant marker for each of the three traits brix, fruit thickness, and fruit width, while no significant markers were found for the rest of the traits. The Manhattan plots for all the traits using GLM model were presented in Figure S4. Consequently, the significant markers that the FarmCPU model identified will be the focus of this study. Figure 2a displays the distribution of all 103 significant single nucleotide polymorphisms (SNPs) across the chromosomes of mango. These markers were covered all the mango chromosomes. The greatest number of significant SNPs (12) was observed on chromosome 3, while the lowest number of significant SNPs (1 SNP) was detected on chromosomes 15. Stone weight had the highest number of significant SNPs (18 SNPs) followed by seed weight (11 SNPs), then fruit weight (9 SNPs). While the lowest number of significant SNPs (2) showed in stone width (Fig. 2b). The Manhattan plot for all morphometric fruit quality traits is presented in Fig. 3. Detailed information on the significant markers associated with these traits using FarmCPU is presented in Table S3, and the significant markers detected using GLM were presented in Table S4. Furthermore, a summary of the significant markers identified for each trait is presented in Table 3. The *p* value varied from 1.25984E-10 to 7.40E-06 for markers NC_058152.1_5147960_G_A and NC_058148.1_13940961_A_G, respectively. The maximum allele effect was observed in marker NC_058142.1_17753741_T_C, which was significantly associated with fruit weight. The lowest allele effect was observed in marker NC_058155.1_12601679_A_T, which was significantly associated with fruit weight.

**Table 3:** GWAS analysis summary for morphometric fruit quality traits describing the range of *p*-values, the range of allele effects, and the number of SNPs linked to these traits

**Trait**	**No. of Markers**	***P* value Range**		**Allele effect Range**	
		**Min.**	**Max**	**Min.**	**Max**
Fruit Weight	9	8.35E-07	7.40E-06	−65.24	80.75
Fruit Length	7	7.71E-08	5.88E-06	−8.62	13.7
Fruit Thickness	4	1.11E-06	5.48E-06	−7.37	5.32
Fruit Width	8	2.61E-10	3.90E-06	−5.33	6.69
Stone Weight	18	3.02E-07	7.34E-06	−5.8	7
Stone Length	6	1.14E-09	3.52E-06	−5.4	11.72
Stone Thickness	6	7.49E-08	5.79E-06	−7	0.91
Stone Width	2	2.18E-06	6.47E-06	−1.32	2.2
Seed Weight	11	2.12E-06	6.77E-06	−4.08	4.89
Seed Length	6	1.44E-09	2.40E-06	−3.52	4.86
Seed Thickness	6	1.26E-10	4.95E-06	−0.96	3.18
Seed Width	6	3.83E-07	6.94E-06	−7.62	3.58
Pulp	7	1.05E-09	4.11E-07	−1.81	2.09
Brix	7	2.49E-09	2.91E-06	−0.87	1.76

### Significant pleiotropic effect markers

The GWAS results revealed that 7 common SNP markers are significantly associated with at least two phenotypic traits suggesting possible pleiotropic effect or indirect effects of these markers on mango morphometric fruit quality traits. Interestingly, these common markers were also detected by the GLM model, which even though did not reveal several markers detected by FarmCPU. Table 4 provides a detailed description of the results for 7 common markers that are supported by various GWAS models. These markers were distributed on 6 chromosomes (3, 4, 7, 17,19 and 20). The lowest *p* value 9.41E-08 was observed for marker NC_058140.1_12343085_C_A, which associated with 3 different traits: fruit weight, fruit thickness and fruit width; this marker was reported in the three different models MLM, GLM and FarmCPU. The largest *p* value 7.34E-06 was observed for marker NC_058139.1_22916155_G_A, which was significantly associated with two different traits, seed length and stone weight, in two models GLM and FarmCPU. The maximum allele effect 8.62 was observed for marker NC_058156.1_9437676_A_G, which was significantly associated with seed weight and seed length in two models. The lowest allele effect − 101.41 was observed for in marker NC_058140.1_12343085_C_A, which was significantly associated with fruit weight, fruit thickness and fruit width in three different models.

**Table 4:** Summary of GWAS results for the common significant markers supported by different GWAS models using 135,079 SNPs

GWAS Model	Associated Traits	Marker ID	Chromosome	Position	Allele Effect	*P-*value
FarmCPU	Seed Length and Stone Weight	NC_058139.1_22916155_G_A	3	22916155	3.44	8.83E-07
					5.58	7.34E-06
GLM	stone weight				5.58	7.34E-06
FarmCPU	Fruit Thickness, Fruit Weight, and Fruit Width	NC_058140.1_12343085_C_A	4	12343085	−7.37	1.11E-06
					−59.01	8.35E-07
					−5.33	9.41E-08
GLM	Fruit Thickness, Fruit weight and Fruit Width				−7.51	6.4E-07
					−101.41	3.4E-06
					−9.55	1.56E-08
MLM	Fruit Thickness and Fruit Width				−7.58	6.43E-06
					−9.25	1.14E-06
FarmCPU	Seed Thickness and Stone Thickness	NC_058143.1_10614972_T_G	7	10614972	−0.96	3.825E-06
					−1.40	7.49E-08
FarmCPU	Stone Weight and Seed Weight	NC_058143.1_9040790_C_T	7	9040790	2.94	2.12E-06
					4.62	2.68E-06
GLM	Stone Weight				4.62	2.68E-06
FarmCPU	Stone Weight and Seed Weight	NC_058153.1_11623544_A_G	17	11623544	−3.70	4.89E-06
					−5.80	6.56E-06
GLM	Stone Weight				−5.80	6.56E-06
FarmCPU	Fruit Width and Fruit Thickness	NC_058155.1_12441789_G_A	19	12441789	−5.08	2.79E-06
					−3.70	6.74E-08
GLM	Fruit Width				−5.83	4.52E-06
FarmCPU	Seed Weigh and Seed Length	NC_058156.1_9437676_A_G	20	9437676	4.86	4.86E-07
					4.89	2.22E-06
GLM	Seed Weigh and Seed Length				8.62	2.39E-06
					5.46	2.46E-06

### Gene annotation of significant markers associated with morphometric fruit quality traits

The candidate genes associated with significant SNPs detected by GWAS for mango morphometric fruit quality traits are presented in Table S3. Gene annotation analyses revealed 87 candidate genes, which belong to different functional groups including different transcriptional factors, amino acids receptors, auxin-induced protein, mitogen-activated protein kinases, serine/threonine-protein kinase, ethylene-responsive transcription factor ERF017, CDPK-related kinase 5-like, transcript variant X2. According to the GO enrichment analysis (Figure S5), the candidate genes in our results are involved in three functional categories: 40 pathways belonging to biological processes, 18 pathways belonging to cellular components, and 18 different pathways belonging to molecular functions. Most of these proteins, enzymes and transcriptional factors had a direct and indirect role in the fruit development stage. As mentioned above, the mitogen-activated protein kinases (MAPKs) family, which is involved in different pathways, plays an effective role in various fruit developmental stage. Interestingly, we detected the MAPKs in the sphingolipid biosynthesis pathway, which plays involved in different roles in plant processes like pollen development, signal transduction and in the response to biotic and abiotic stress. MAPKs are involved in hormonal signaling pathway (Figure S6). Three genes, LOC123211358, LOC123220493 and LOC123200811 code for serine/threonine-protein kinase. LOC123211358 was found to be associated with stone weight and stone width while LOC123220493 was associated with stone thickness and LOC123200811 was found to be associated with seed stone length.

One significant marker NC_058140.1_12343085_C_A, supported by three different GWAS model, was associated with fruit weight, fruit thickness and fruit width. This marker was annotated to LOC123212704. which codes for a calcium‑dependent protein kinase (CDPK).

### Promising mango genotypes for future breeding program

**Fig. 4:**
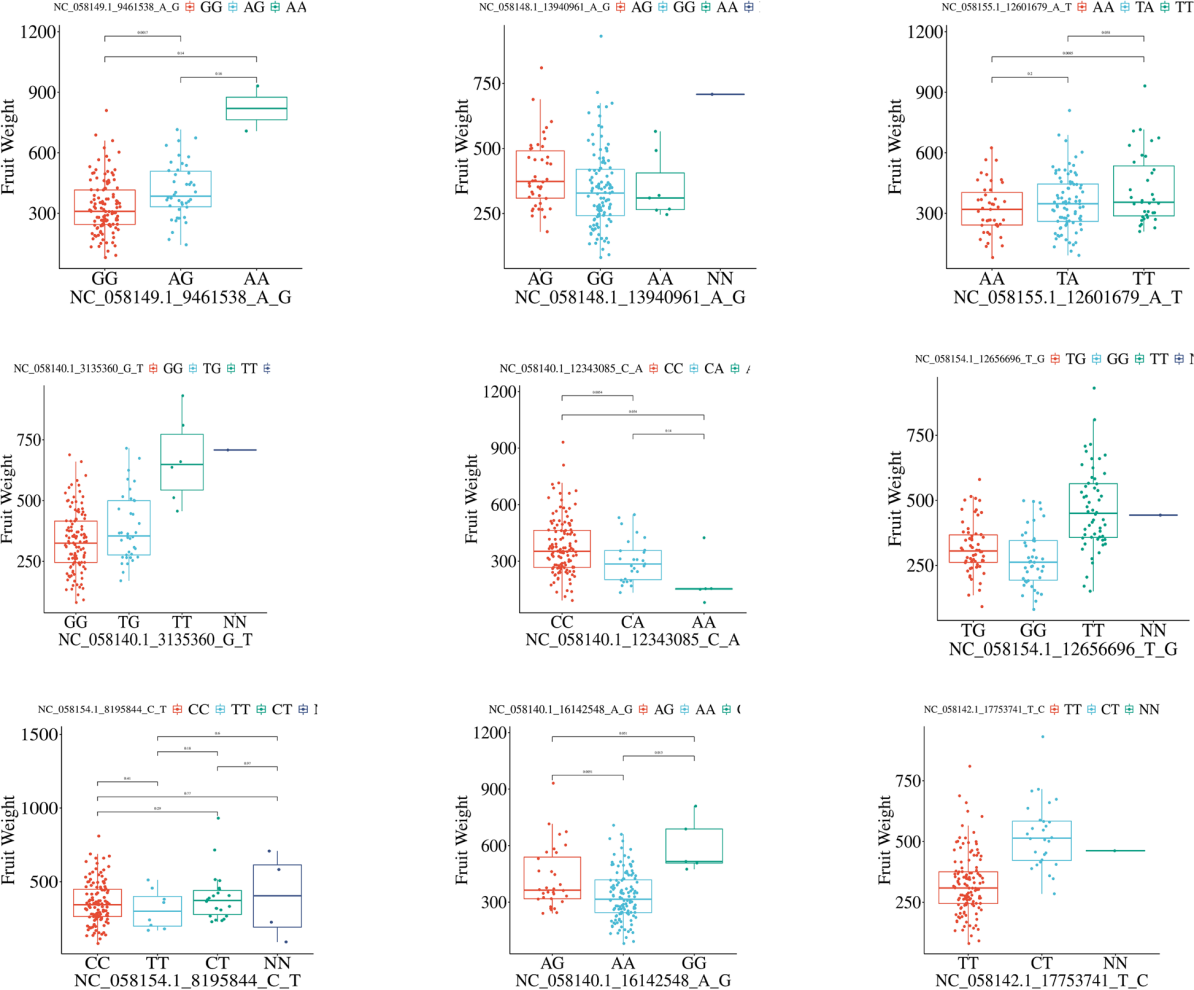
Box plots showing the distribution of reference allele, alternative allele and heterozygote allele among 161 mango accessions for each significant marker associated with fruit weight

**Fig. 5:**
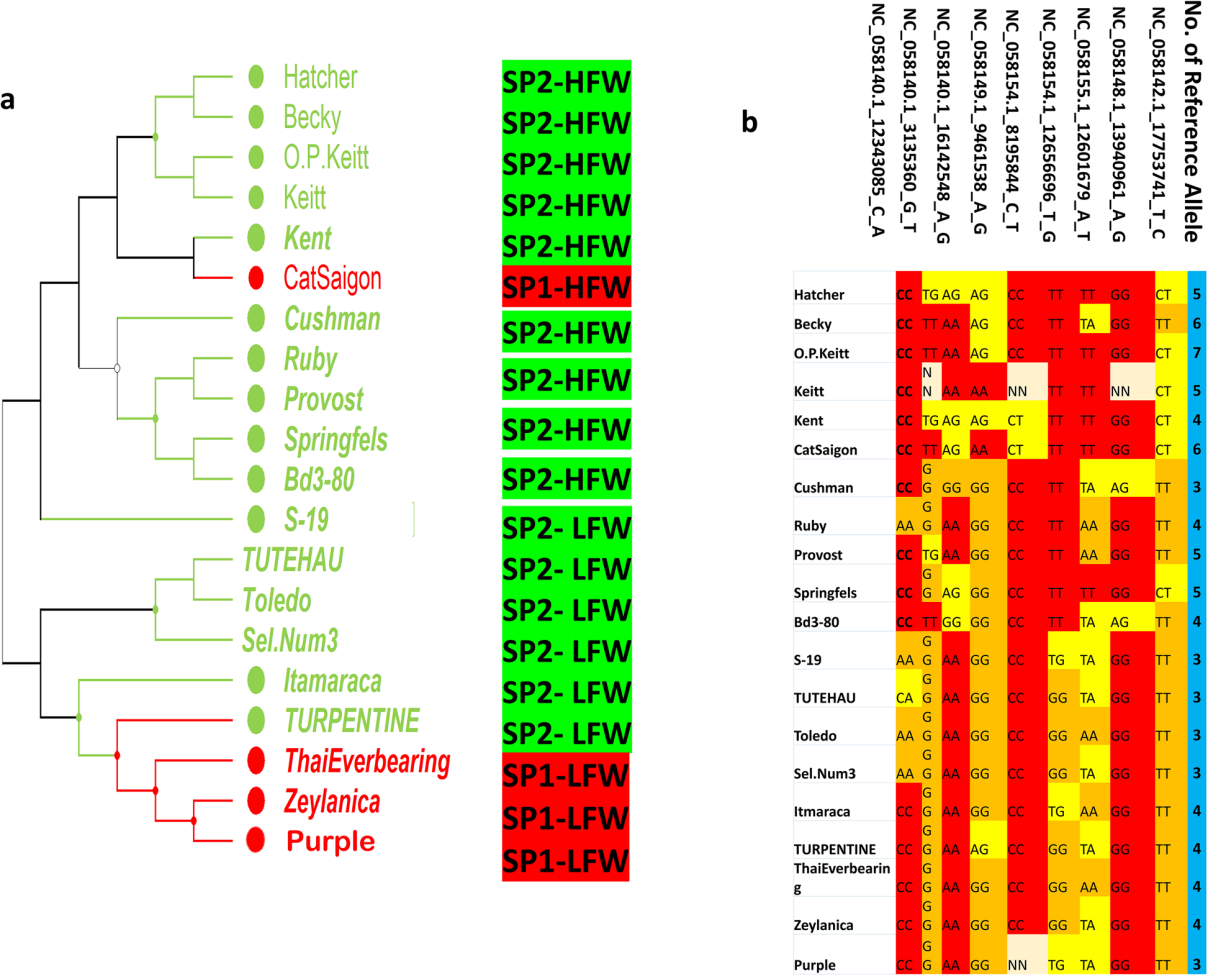
The hierarchical cluster analysis of 20 selected mango accessions based on fruit weight. **a **The accession name with red color refers to SP1, green color refers to SP2. “H-FW” denotes accessions belonging to the highest fruit weight group, while “LFW” indicates those belonging to the lowest fruit weight. **b** The allele matrix of 9 SNPs associated with fruit weight. Red color refers to homozygous allele for large fruit weight, orange color refers to homozygous allele for small fruit weight, yellow color refers to heterozygous allele, blue column shows number of reference alleles

In order to improve our findings, we focused on mango fruit weight because it is thought to be the most appealing trait for the commercial market, and it has the highest phenotypic variation among our accessions. Our analysis revealed nine significant markers associated with fruit weight. These markers are located on different chromosomes, including chromosomes 4, 6, 12, 13, 18, and 19. Figure 4 presents the distribution of the reference allele, alternative allele, and heterozygote allele for each significant marker among all the mango accessions, providing information on the allele that regulates this important trait. All the markers in our mango collection displayed three functional alleles, except for one marker, NC_058142.1_17753741_T_C, which exhibited both a heterozygote allele (TC) and one of the homozygous allele (TT). The homozygous allele with a high median in the box plots is considered the reference allele for fruit weight. To increase the genetic diversity in our germplasm collection, twenty mango accessions were selected, ten with high and ten with low fruit weight. Fruit weight varied between 80.71g and 154.41g for the low- fruit weight accessions and between 624.76g and 931.33g for the high-fruit weight accessions. The genetic distance among the 20 accessions is illustrated in dendrogram cluster analysis (Fig. 5a). All 20 accesions were from the two subpopulations (SP) according to our population structure analysis with 4 and 16 belonging to SP1 and SP2, respectively. The genetic distance ranged from 0.15 (‘Hatcher’ and ‘Kiett’; and ‘Hatcher’ and “Kent’) to 0.34 (‘ThaiEverbearing’ and ‘Becky’; and ‘Kent’ and ‘Purple’) (Table S5). Figure 5b displays the distribution and total count of reference alleles in each selected accession. The accession 'O.P. Kiett' had the largest number of reference alleles, with a count of 7 allele. This was followed by two different accessions, 'Becky' and 'CatSaigon', which both had 6 reference alleles. These data are reflective of the phenotypic results we have achieved over a period of nine years. These cultivars are classified as having a high fruit weight. The weight of the mango fruit for 'O. P Kiett' was 636.97g, whereas the weight of the 'Becky' mango fruit was 659.92g, and the weight of the 'CatSaigon' mango fruit was 931.33g. The accessions 'S_19', 'TUTEHAU', 'Toledo', 'Sel.Num3', and 'Purple' had the lowest count of reference alleles, which was 3 alleles. Using the results derived from genetic distance between accessions, GWAS analysis, population structure and our understanding of the presence of reference alleles controlling this trait, we could enhance the genetic diversity of our mango germplasm by crossing accessions with high fruit weight and accessions with small fruit weight. To gather as many genes as possible that control fruit weight, we highly recommend crosses between ‘Becky’ × ‘Thai Everbearing’ and ‘Kent’ × ‘Purple’.

## Discussion

In the present study, we conducted GWAS to identify SNP markers associated with 14 different mango fruit traits using a panel of 161 accessions. Since these accessions originated from different geographic regions and carry substantial genetic diversity, it is expected that a major fraction of the phenotypic diversity in fruit quality traits is likely attributed to genetic factors, in addition to environmental factors. To accelerate the improvement of fruit traits in mango varieties, this panel of 161 accessions provides an opportunity to discovery novel quantitative trait loci (QTLs) and markers for application in in mango breeding programs. Genetic diversity arises from evolutionary processes such as mutation, migration, and genetic drift, which drive shifts in allelic frequency that may cause statistical associations between random genetic markers and traits [46, 47]. However, this genetic structure-related variation often needs to be clarified with loci contributing to trait variation in association studies, resulting in an inaccurate representation of the genotype to phenotype map predicted by genomic selection models [48–51]. It is crucial to carefully account for the underlying genetic structure in association studies to avoid such errors. Studies such as this one, employing rigor and precision in their methodology, are essential for advancing our understanding of the genetic basis of complex traits. Studying genetic variation and population structure is crucial in understanding mango improvement and marker-trait association research. Indian mango cultivars, which are geographically diverse, have served as a genetic foundation for local mango cultivation in other parts of the world [52]. The results of our study revealed that mango accessions can be divided into two groups based on population structure and PCA analysis. The first group, SP1 in Fig. 1b, comprises most Indian and Southeast Asian accessions in one cluster. The second group, SP2, includes accessions from the USA, South America, and Caribbean Countries. These findings align with the current understanding that mangoes originated in Northeast India and spread eastward, primarily polyembryonic, and westward, primarily monoembryonic [9, 52–54]. Our STRUCTURE analyses, supported by PCA, also identified several admixed accessions that resulted from natural crossing, gene flow, and genetic drift. These findings are consistent with previous studies, such as that of Ravishankar et al., [55], who assessed the genetic diversity and population structure of mango accessions from India using simple sequence repeat markers. Their model-based structural analysis revealed that cultivars from the "Southwest" and "Northeast" regions were part of two subpopulations. Similarly, Sherman et al., [54] found the same results using 289 high-quality SNP data to genotype 74 accessions of the Israeli mango collection. The mango collection was divided into two major groups: one consisting primarily of Indian accessions clustered together with accessions from Southeast Asia, which corresponds to SP1 in our analyses, and a larger group consisting of Floridian, South African, Australian, Israeli, and South American accessions, which corresponds to SP2.

### Genome wide association mapping reveals novel QTLs in morphometric fruit quality traits

The discovery and implementation of associations between molecular markers and fruit traits are expected to expedite fruit tree cultivars' breeding significantly. The current study presents the first Genome-wide association study (GWAS) of mango morphometric traits using high-quality SNP markers. In GWAS, various models are employed to increase the statistical power of identified markers, reduce false favorable rates caused by population structure and kinship, and minimize confounding effects related to population structuring [56]. Typically, every marker is tested for association with a trait, which can lead to false positives and inflation of test statistics due to the inclusion of population structure and kinship information. Using mixed linear models and principal component analyses can reduce the inflation of test statistics and confounding due to population structure, albeit only for small-effect loci [57, 58]. Multi-locus mixed linear models (MLMLM) are widely used to address large-effect loci and reduce confounding effects. However, these models still need to address these issues thoroughly.

To overcome these challenges, fixed and random model circulating probability unification (FarmCPU) divides the MLMLM into fixed and random effect models and conducts them iteratively [59]. This approach has been shown to improve the statistical power of the results and computational efficiency. FarmCPU is computationally efficient because the marker testing is conducted by a fixed effect model (FEM), which has a computing time complexity linear to the number of markers and individuals. Additionally, test statistics *P*-values for non-pseudo–quantitative trait nucleotides (QTN) markers are not inflated [44]. The model includes all markers that impact a phenotype as either pseudo QTNs or markers associated with pseudo QTNs. Significant *P*-values for non-pseudo QTN markers are not expected because association tests on all markers are carried out with pseudo QTNs as covariates [44]. The FarmCPU model was employed using 135,079 high-quality SNPs to uncover the genetic basis of 14 morphometric fruit quality traits in the present study.

A total of 103 significant SNP markers were associated with 14 traits, some of which were unique for each trait, and others were common, impacting at least two different traits. Since the fruit traits analyzed in this report are quantitative, each is impacted by multiple loci with additive contributions. This was evident in our analyses, showing that most loci had minor effects and can be considered minor QTLs. Although QTL mapping and development of molecular markers are scarce in mango, in a recent study, using 80K SNPs QTLs were mapped for fruit firmness and fruit color in a bi-parental population [20]. Similarly, in a prior study, Azam et al., [16] identified markers associated with 16 phenotypic traits, including fruit weight, fruit width, and brix, by employing 17 simple sequence repeat (SSR) loci. Similarly, Padmakar et al., [15] investigated 31 SSR markers on various fruit traits, including fruit weight and pulp content, in a 48-mango core collection and found one marker associated with fruit weight. In contrast to these works, we employed high-quality single nucleotide polymorphism (SNP) markers, identifying significantly associated markers with fruit quality traits. The annotation of genes near these significant markers revealed different proteins, enzymes, and transcription factors involved in various pathways. The traits reported in this study heavily influence the development, maturity, and ripening process of mangoes. Plant hormones, growth regulators, and various biological and environmental variables interact extensively during fruit development and ripening. The ripening of fleshy fruits such as mangoes involves ethylene production, chloroplast differentiation, pigment accumulation (carotene and lycopene), flavor and aroma development, and softening of fruit tissues [60, 61].

Our findings suggest that three genes, LOC123211358, LOC123220493 and LOC123200811, which encode mitogen-activated protein kinases (MAPKs). Mitogen-activated protein kinases (MAPKs) are integral to various cellular processes including fruit development and fruit size traits. Studies provided by [62, 63] have shown that MAPK cascades are involved in the regulation of grain size and spikelet number per panicle in rice, suggesting a similar mechanism could be at play in other fruit-bearing plants. Also, MAPKs might be involved in fruit ripening, as MAPK cascades are implicated in plant fruit development via an ethylene signaling pathway [25, 64]. Also, the role of 10 MAPKK and 77 MAPKKK genes in tissue development, fruit growth and ripening, and response to abiotic stressors such cold, drought, and salt was reported by [26] based on a full transcriptome of bananas. Li et al., [64] found that a transcriptional study of the cultivated strawberry (*Fragaria* × *ananassa*) revealed the expression of all FaMAPK genes at every developmental stage. They observed that the expression of FaMAPK genes augmented endogenous abscisic acid (ABA), sucrose, and anthocyanin levels in strawberry fruits. This finding implied a close relationship between MAPK genes and strawberry ripening. The similarity of our study's MAPK genes to the ones discovered in the published reports suggests that comparable pathways affect fruit quality traits in diverse plant species. The MAPK signaling cascade, which consists of varying number of MAPKs, MAPKKs and MAPKKs, is highly conserved in higher plants with *Arabidopsis* harboring 20 MAPKs, 10 MAPKKs, and 80 MAPKKKs [24, 65], rice 17 MAPKs, 8 MAPKKs, and 75 MAPKKKs [66, 67], maize 19 MAPKs, 9 MAPKKs, and 71 MAPKKKs [68, 69], tomato 16 MAPKs, 6 MAPKKs, and 89 MAPKKK genes [70, 71], and cucumber harboring 14 MAPKs, 6 MAPKKs, and 59 MAPKKKs [72]. According to Asif,, et al., [73], a total of 25 MAPKs were identified to play a role in various plant processes.

## Conclusion

In summary, our study has reported 103 significant SNP markers for 14 morphometric fruit quality traits in a collection of 161 mango accessions. Of these, 7 common markers were significantly associated with at least two traits, while 96 unique markers were associated with only one trait. These findings establish a foundation for future studies, enabling precise mapping and identification of candidate genes for the traits investigated in this study. This will serve as a valuable resource for further characterizing novel quantitative trait loci. Furthermore, the significant SNPs identified can be used to investigate genomic selection methods with greater prediction accuracy for fruit traits in mangoes. Additionally, we combined information from genetic diversity, population structure, genetic distance, and GWAS results to identify the most promising mango accessions as potential parents for future breeding programs. Consequently, we identified 20 mango accessions that could be crossed in various combinations to mix diverse genes controlling critical fruit traits. Conducting targeted crosses informed by genetic analyses will be impactful in closing genetic gaps among the accessions maintained at the USDA National Plant Germplasm System. This, in turn, will be useful in developing modern mango cultivars that possess desired horticultural, nutritional, and climate adaptability traits.

## Supplementary Information

Supplementary Material 1: Table S1. List of 161 Mango genotypes, subpopulation relationships and 14 morphometric fruit quality traits and the Shapiro-Wilk test for each trait. Table S2. List of high-quality 135,079 SNPs marker for 161 mango accession. Table S3. Detailed GWAS results for the 14 morphometric fruit quality traits using FarmCPU model with 135,079 SNPs, Gene ID, and the encoding product. Table S4. Detailed GWAS results for the 14 morphometric fruit quality traits using GLM model with 135,079 SNPs, Gene ID, and the encoding product. Table S5. The genetic distance among the 20 selected accession using 135,079 SNP markers.

Supplementary Material 2: Figure S1. Phenotypic distribution of 14 morphometric fruit quality traits in the 161 mango accessions showed the normal frequency distribution for all the traits. Figure S2. Phenotypic correlation analysis among the 14 morphometric fruit quality traits showed the high correlation between most of the traits. Figure S3. QQ plots for all traits scored in three different GWAS model FarmCPU, GLM and MLM. Figure S4. Manhattan plots displaying SNP marker-trait association identified for 14 morphometric fruit quality traits using GLM GWAS model with 135,079 SNPs markers. Figure S5. Gene Ontology (GO) enrichment analysis of mango fruit quality traits. It depicts the results of GO enrichment analysis (BP: biological process, CC: cellular components, MF: molecular function category) using Fisher’s exact test. Each line represents term enrichment, with *p*-values indicating statistical significance displayed along a gradient color from red (less significant) to blue (most significant). Line length corresponds to the count of differentially expressed genes belonging to each term; The y-axis represents enriched GO term. While the x-axis displays the Gene ratio (#significant genes/#annotated genes). Figure S6. The KEGG sphingolipid singling pathway and plant hormonal signaling pathway which included MAPK.

## Data Availability

Sequence datasets analyzed during the current study are available in the https://www.ncbi.nlm.nih.gov/srarepository, under the BioProject ID: PRJNA1132966.
