# Supplementary material for: A genome-wide association study identified SNP markers and candidate genes associated with morphometric fruit quality traits in mangoes
Source: BMC Genomics. 2025 Feb 7;26:120. doi: 10.1186/s12864-025-11278-6 (PMC11806778; doi:10.1186/s12864-025-11278-6)
Supplement: Supplementary file 2 — Supplementary Material 2: Figure S1. Phenotypic distribution of 14 morphometric fruit quality traits in the 161 mango accessions showed the normal frequency distribution for all the traits. Figure S2. Phenotypic correlation analysis among the 14 morphometric fruit quality traits showed the high correlation between most of the traits. Figure S3. QQ plots for all traits scored in three different GWAS model FarmCPU, GLM and MLM. Figure S4. Manhattan plots displaying SNP marker-trait association identified for 14 morphometric fruit quality traits using GLM GWAS model with 135,079 SNPs markers. Figure S5. Gene Ontology (GO) enrichment analysis of mango fruit quality traits. It depicts the results of GO enrichment analysis (BP: biological process, CC: cellular components, MF: molecular function category) using Fisher’s exact test. Each line represents term enrichment, with p-values indicating statistical significance displayed along a gradient color from red (less significant) to blue (most significant). Line length corresponds to the count of differentially expressed genes belonging to each term; The y-axis represents enriched GO term. While the x-axis displays the Gene ratio (#significant genes/#annotated genes). Figure S6. The KEGG sphingolipid singling pathway and plant hormonal signaling pathway which included MAPK. [file 12864_2025_11278_MOESM2_ESM.pdf]

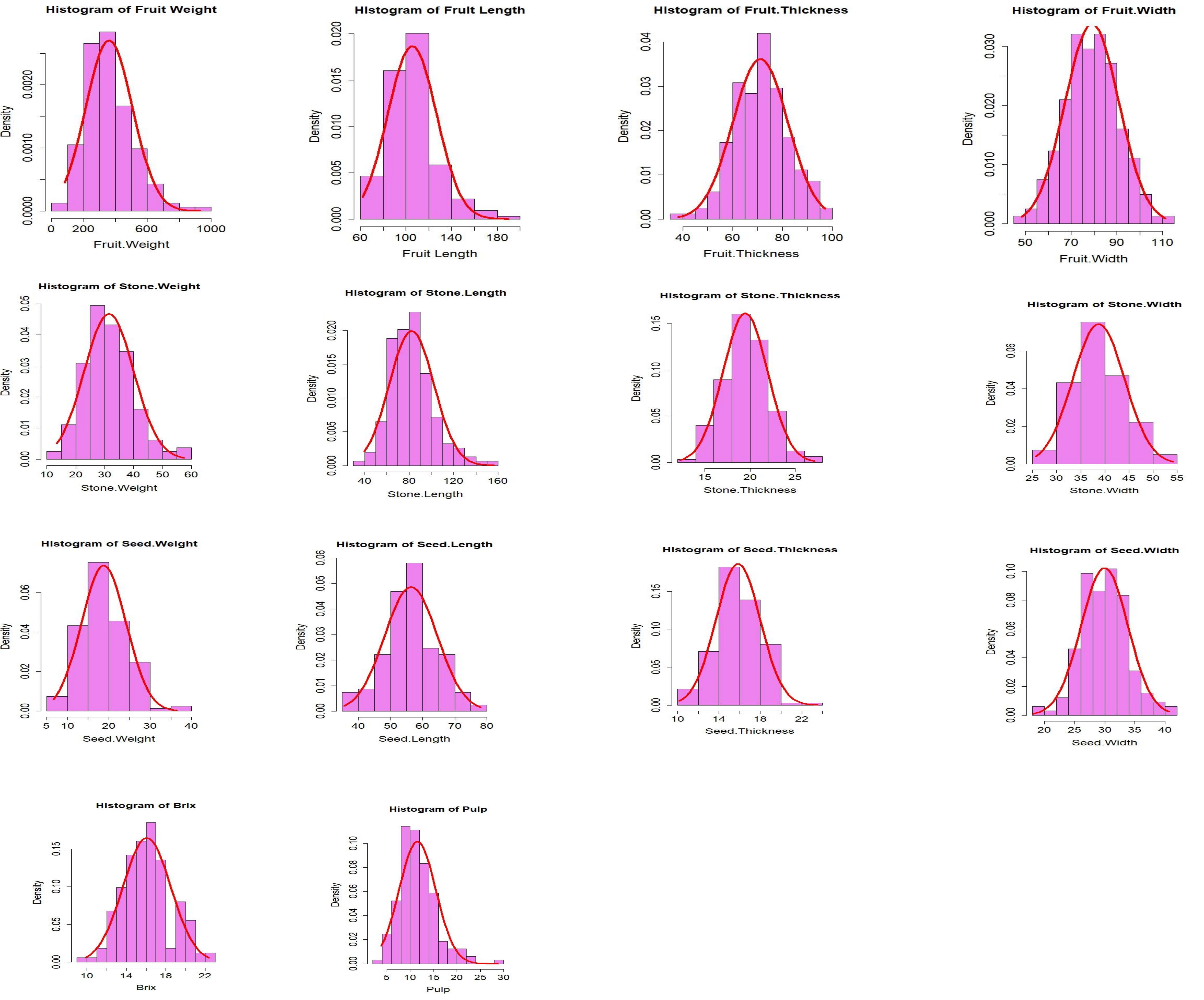

Figure S1: Phenotypic distribution of 14 morphometric fruit quality traits in the 161 mango accessions showed the normal frequency distribution for all the traits

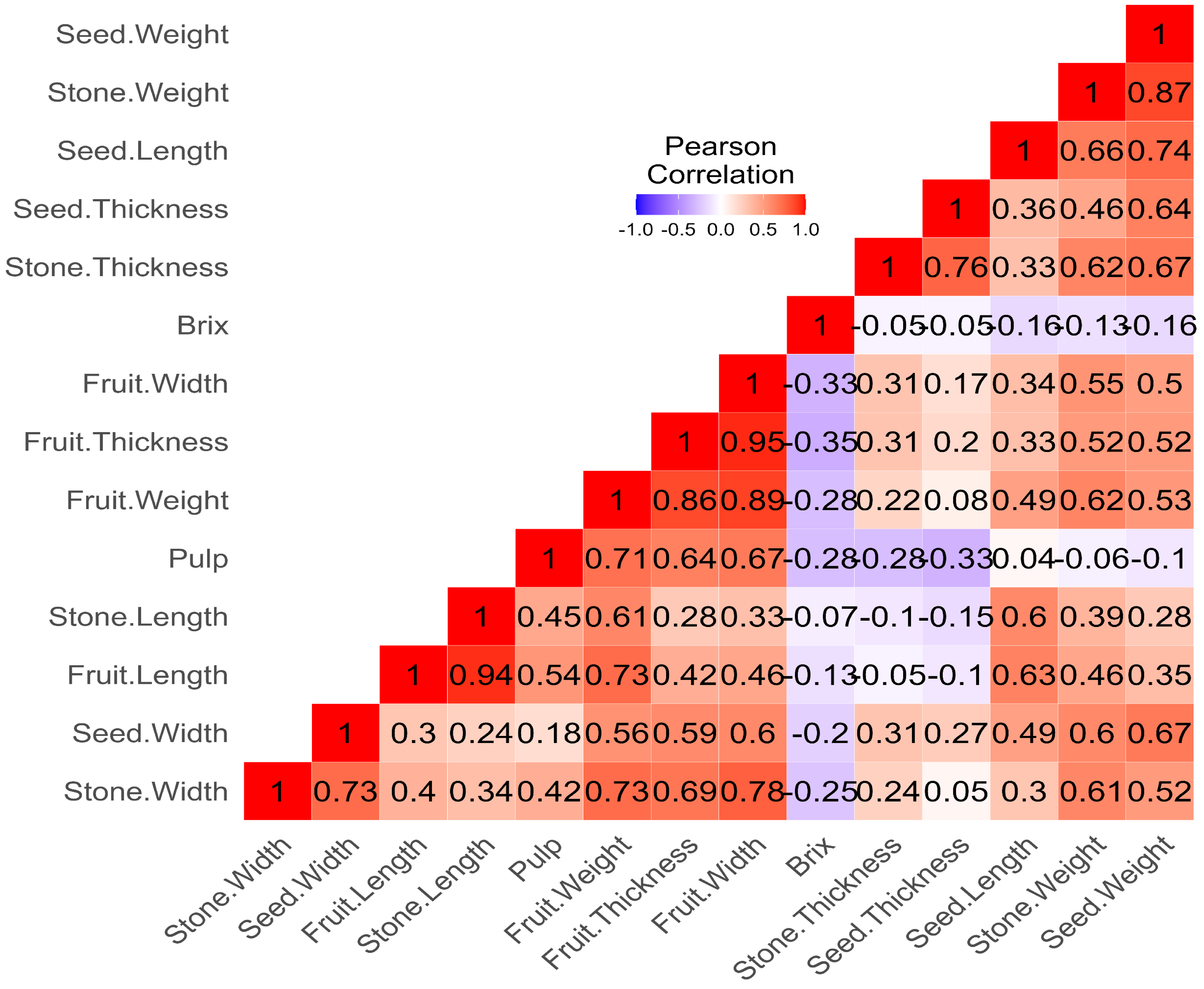

Figure S2: Phenotypic correlation analysis among the 14 morphometric fruit quality traits showed the high correlation between most of the traits.

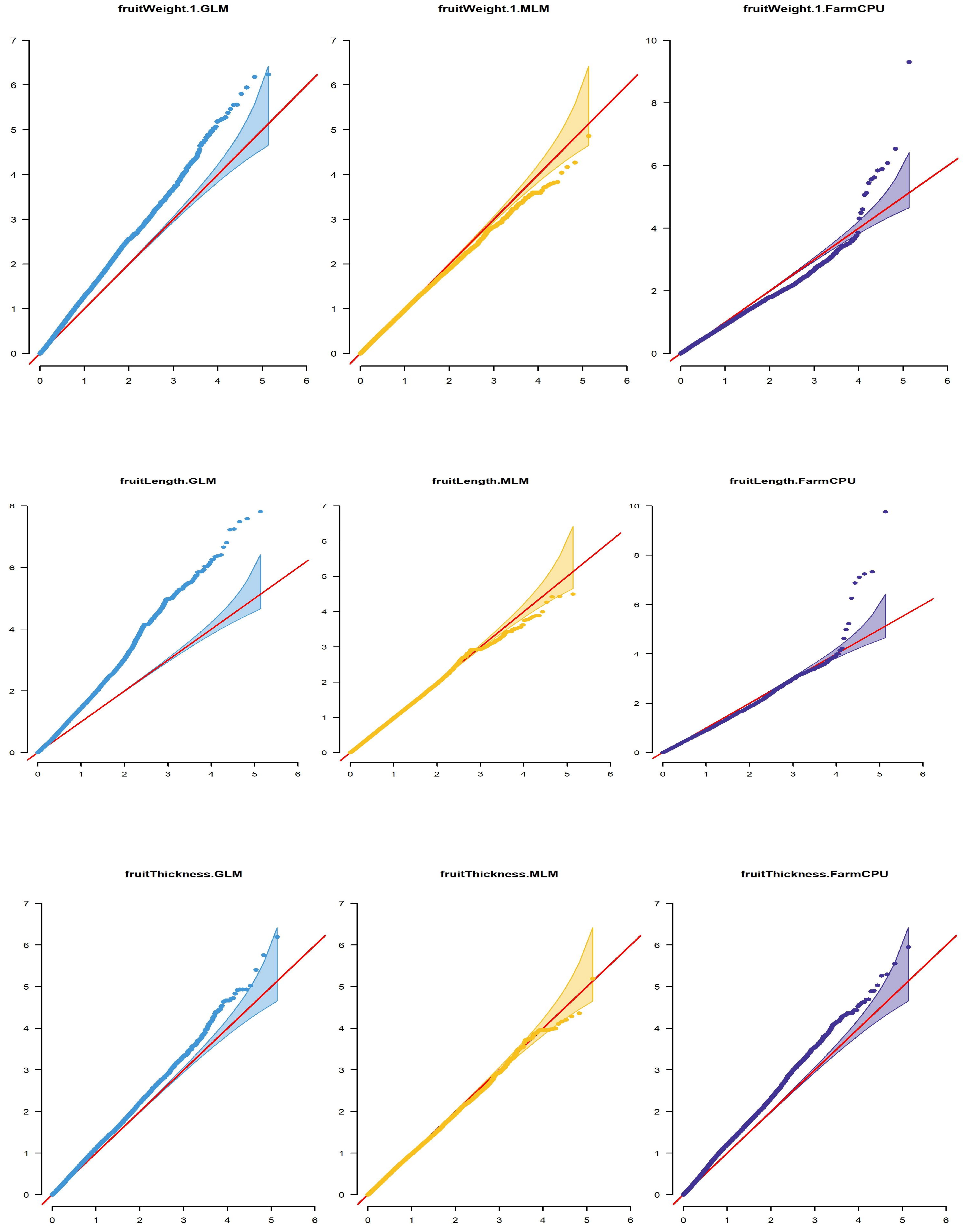
fruitWeight.1.GLM
fruitWeight.1.MLM
fruitWeight.1.FarmCPU
fruitLength.GLM
fruitLength.MLM
fruitLength.FarmCPU
fruitThickness.GLM
fruitThickness.MLM
fruitThickness.FarmCPU

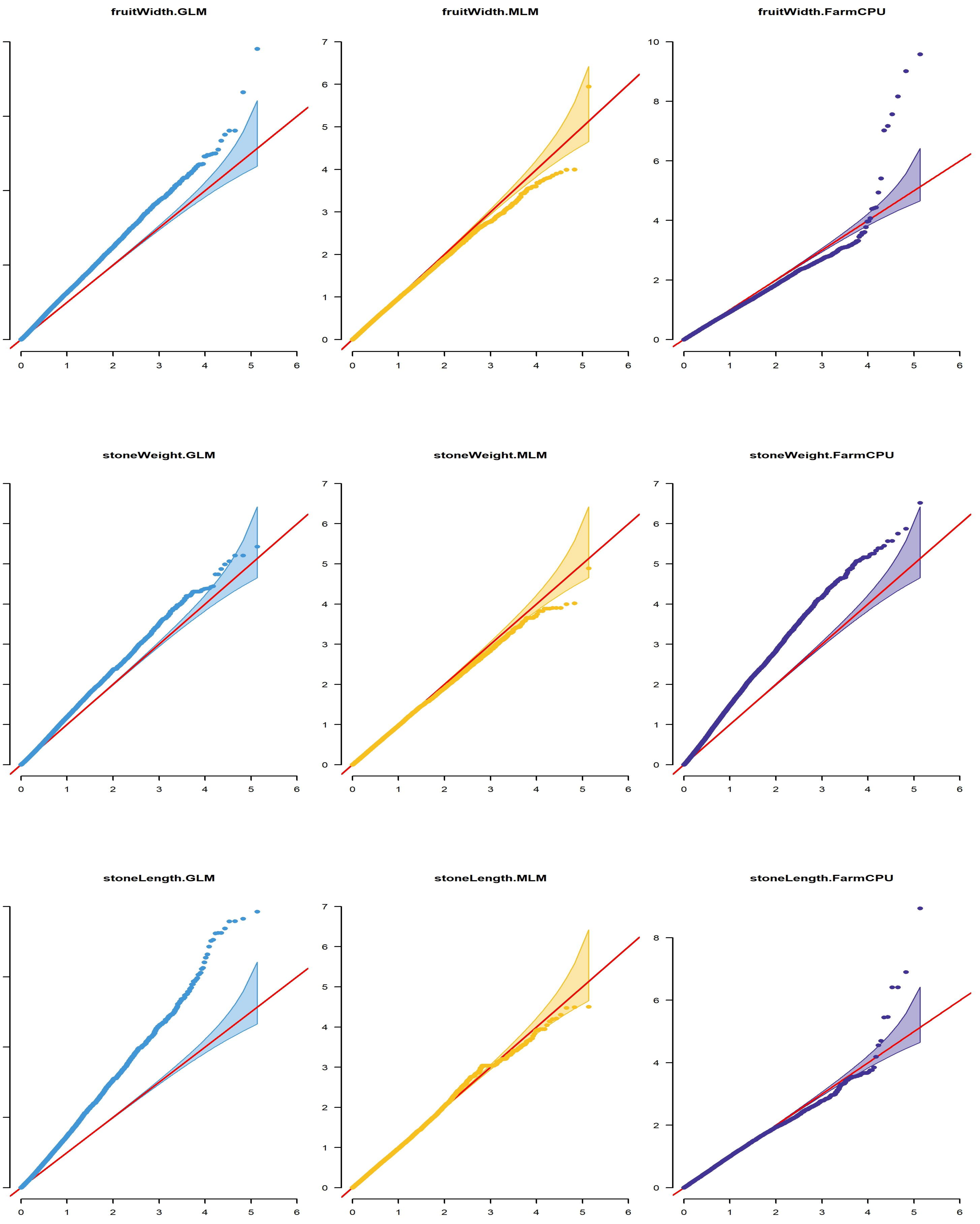
fruitWidth.GLM
fruitWidth.MLM
fruitWidth.FarmCPU
stoneWeight.GLM
stoneWeight.MLM
stoneWeight.FarmCPU
stoneLength.GLM
stoneLength.MLM
stoneLength.FarmCPU

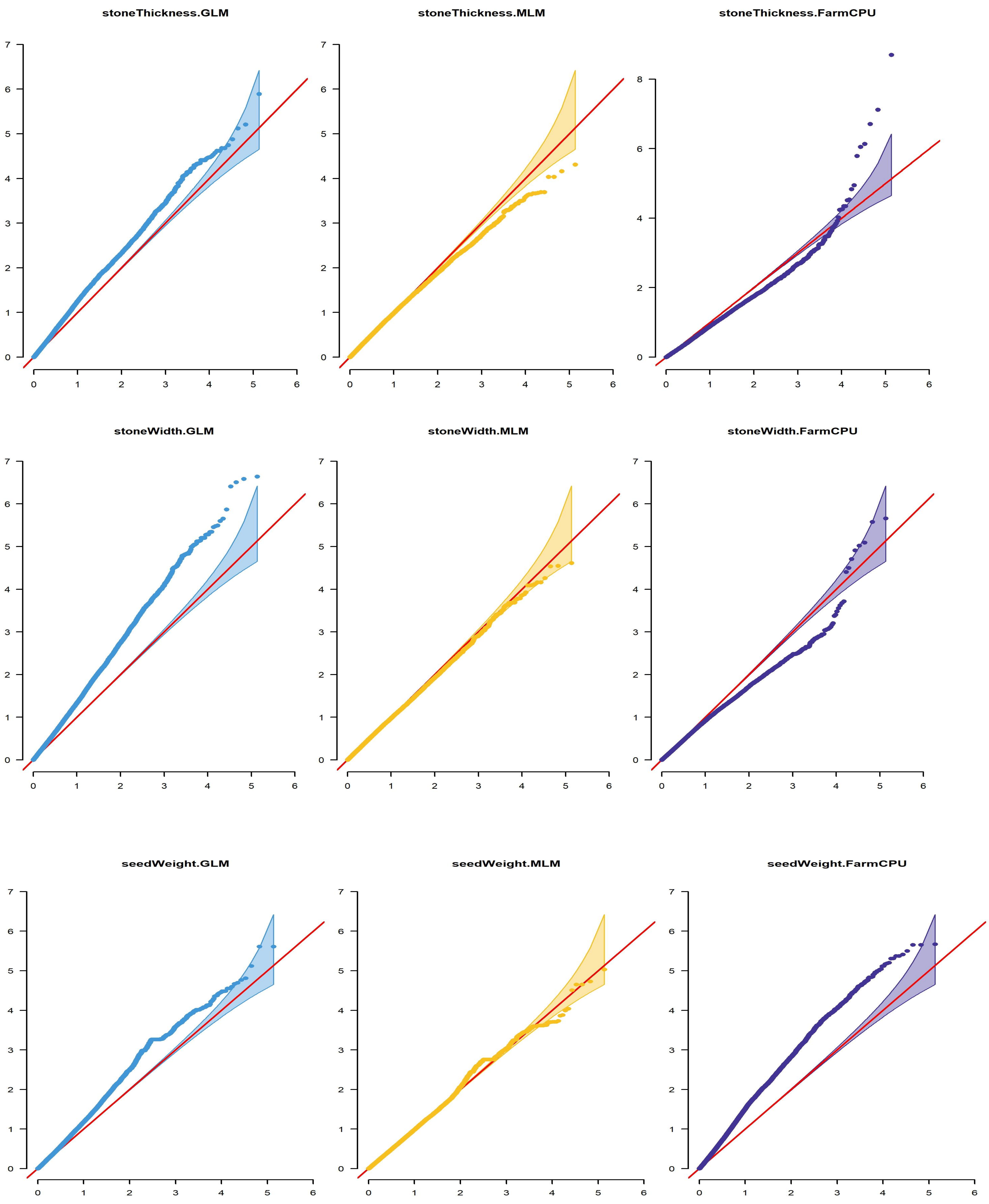
stoneThickness.GLM
stoneThickness.MLM
stoneThickness.FarmCPU
stoneWidth.GLM
stoneWidth.MLM
stoneWidth.FarmCPU
seedWeight.GLM
seedWeight.MLM
seedWeight.FarmCPU
0 1 2 3 4 5 6
0 1 2 3 4 5 6 7
0 2 4 6 8

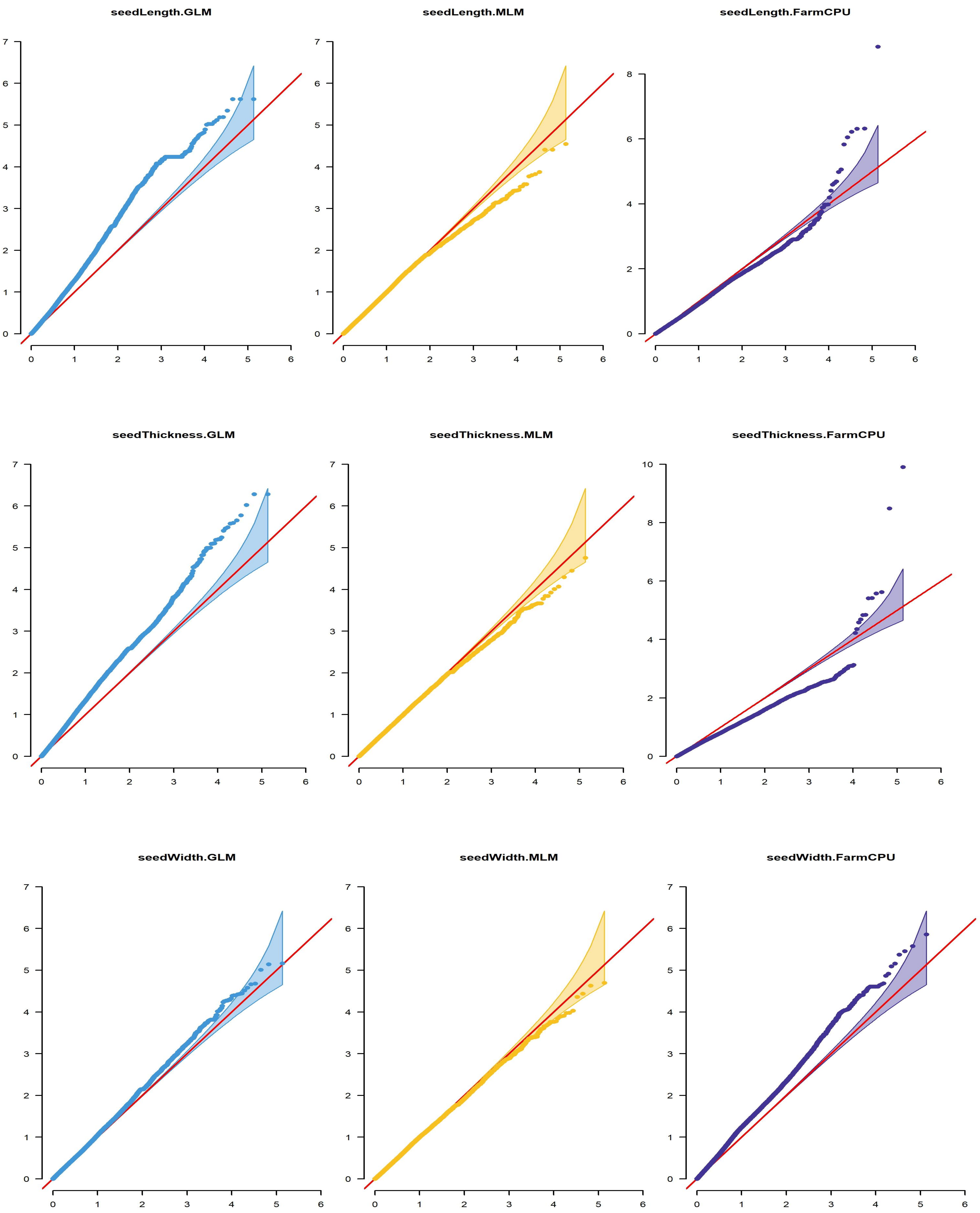
seedLength.GLM
seedLength.MLM
seedLength.FarmCPU
seedThickness.GLM
seedThickness.MLM
seedThickness.FarmCPU
seedWidth.GLM
seedWidth.MLM
seedWidth.FarmCPU

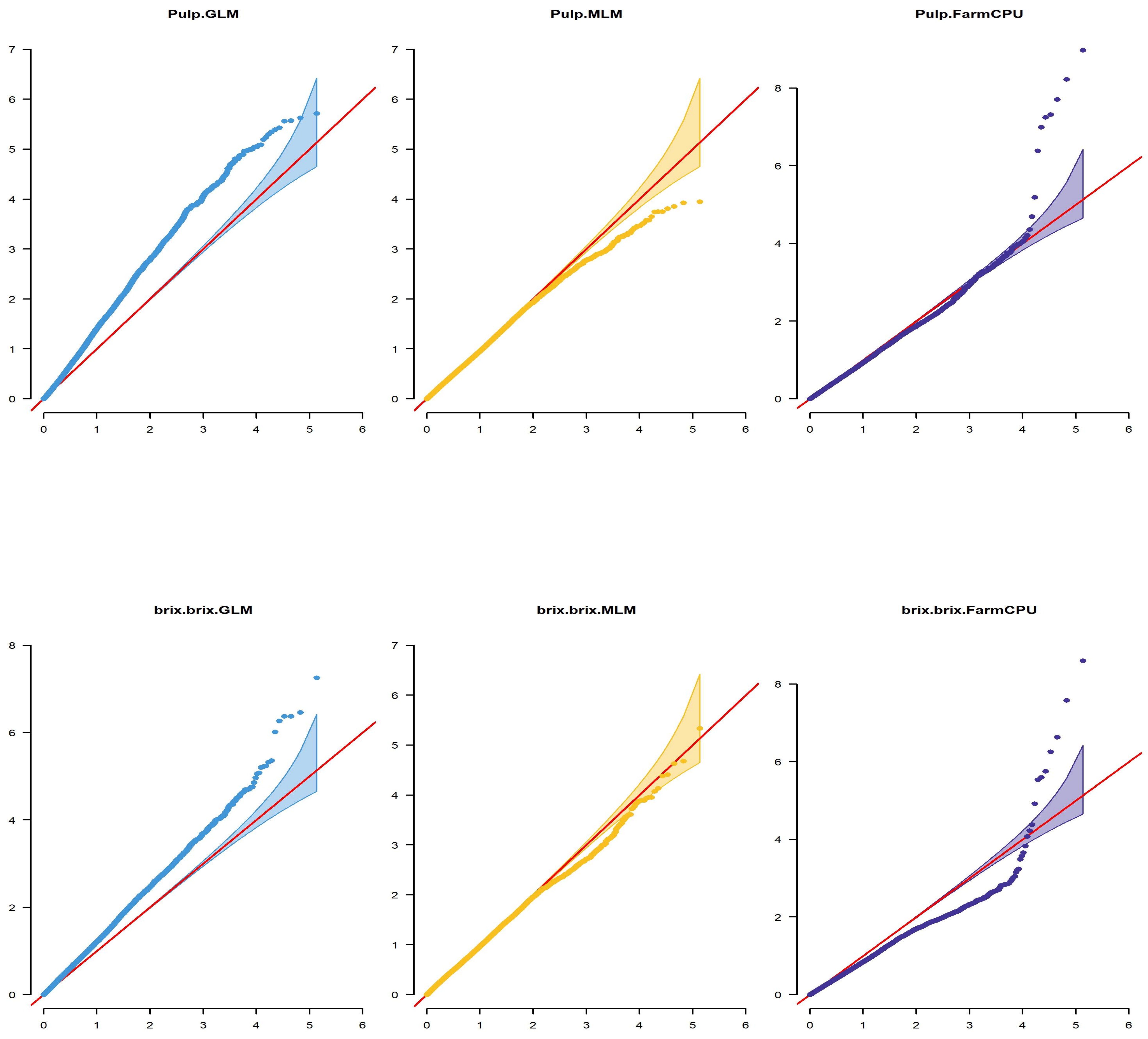

Figure S3. QQ plots for all traits scored in three different GWAS model FarmCPU, GLM and MLM

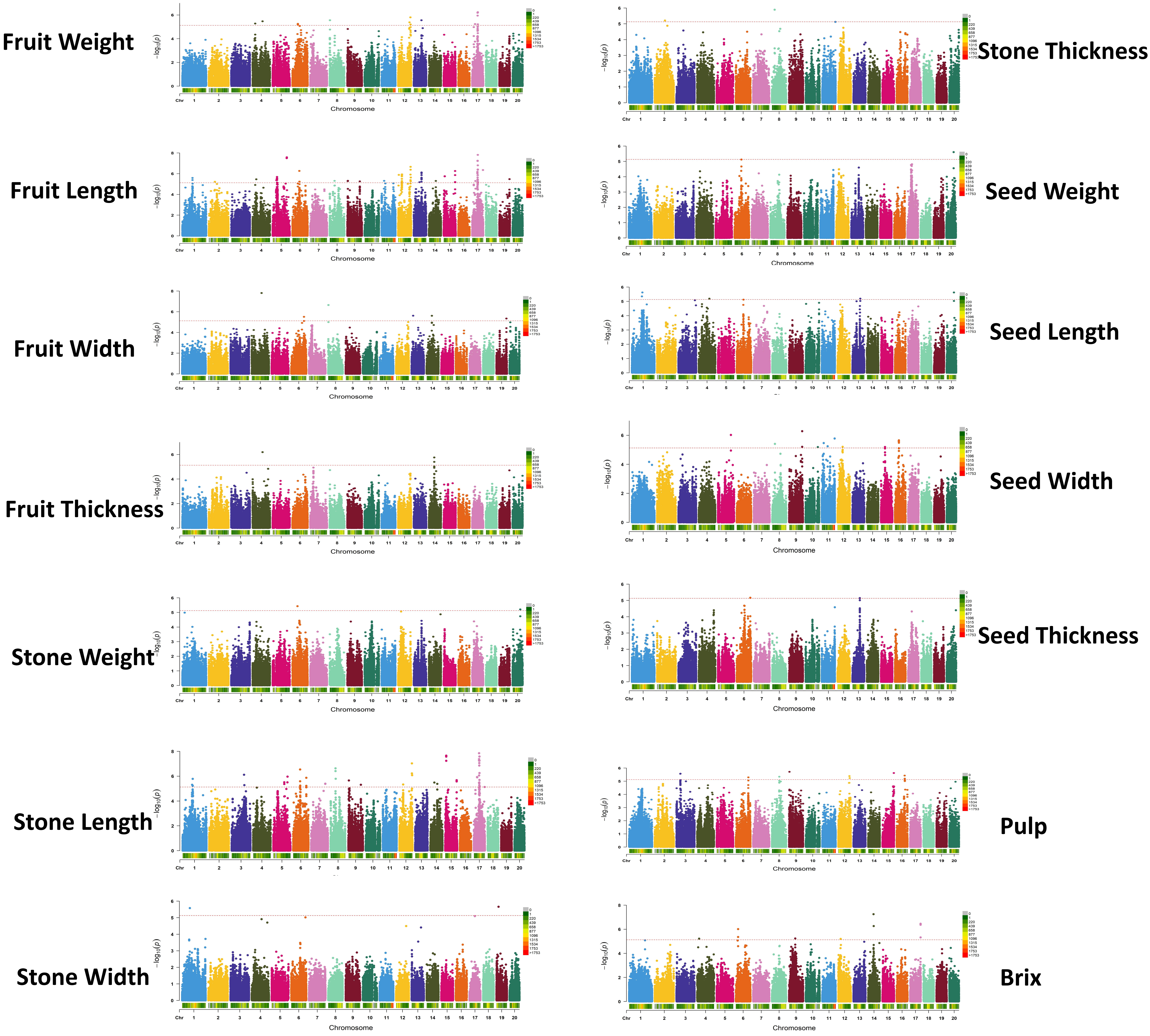

Figure S4. Manhattan plots displaying SNP marker-trait association identified for 14 morphometric fruit quality traits using GLM GWAS model with 135,079 SNPs markers

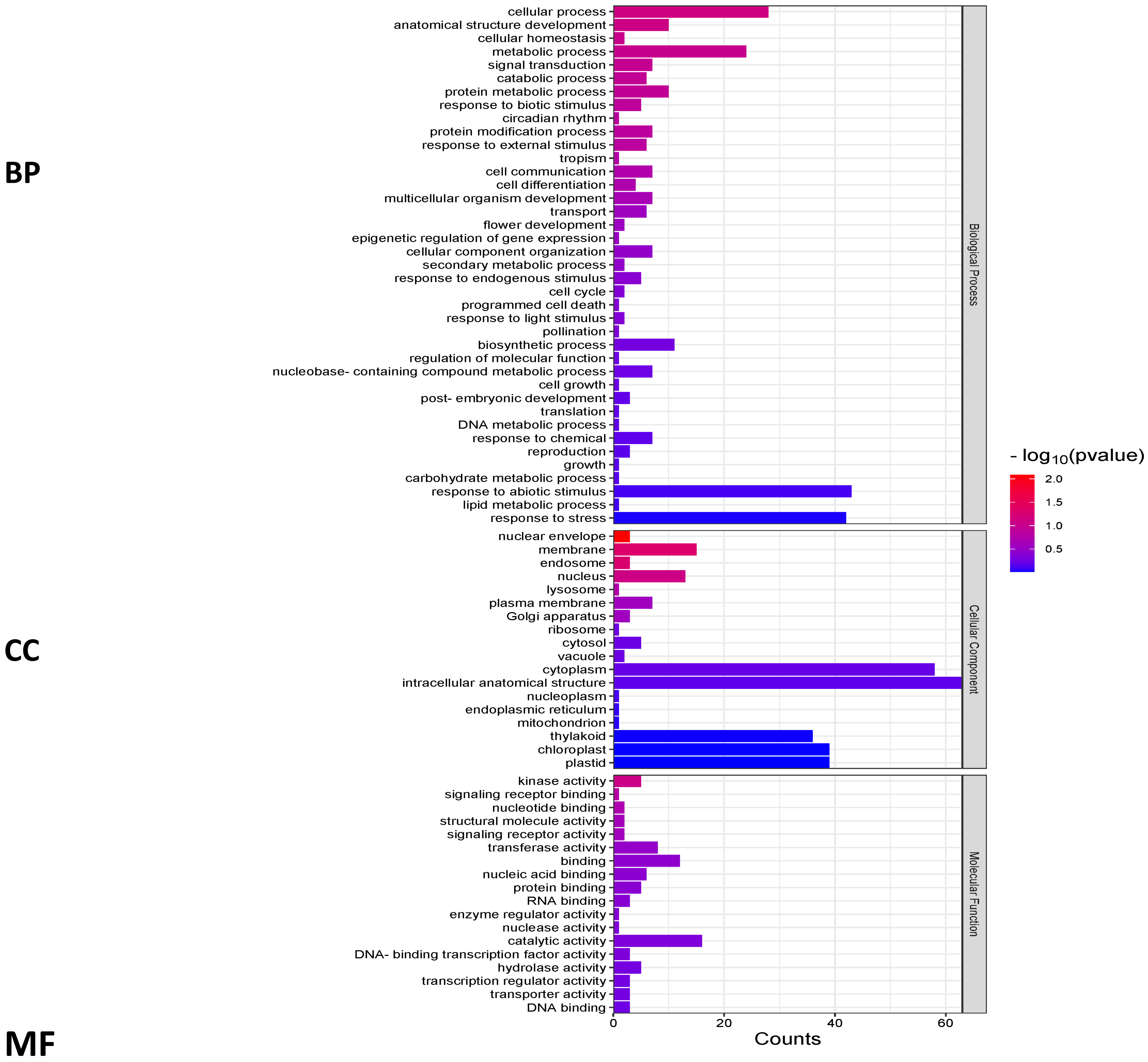

Figure S5: **Gene Ontology (GO) enrichment analysis of mango fruit quality traits.** It depicts the results of GO enrichment analysis (BP: biological process, CC: cellular components, MF: molecular function category) using Fisher's exact test. Each line represents term enrichment, with p-values indicating statistical significance displayed along a gradient color from red (less significant) to blue (most significant). Line length corresponds to the count of differentially expressed genes belonging to each term; The y-axis represents enriched GO term. While the x-axis displays the Gene ratio (#significant genes/#annotated genes).

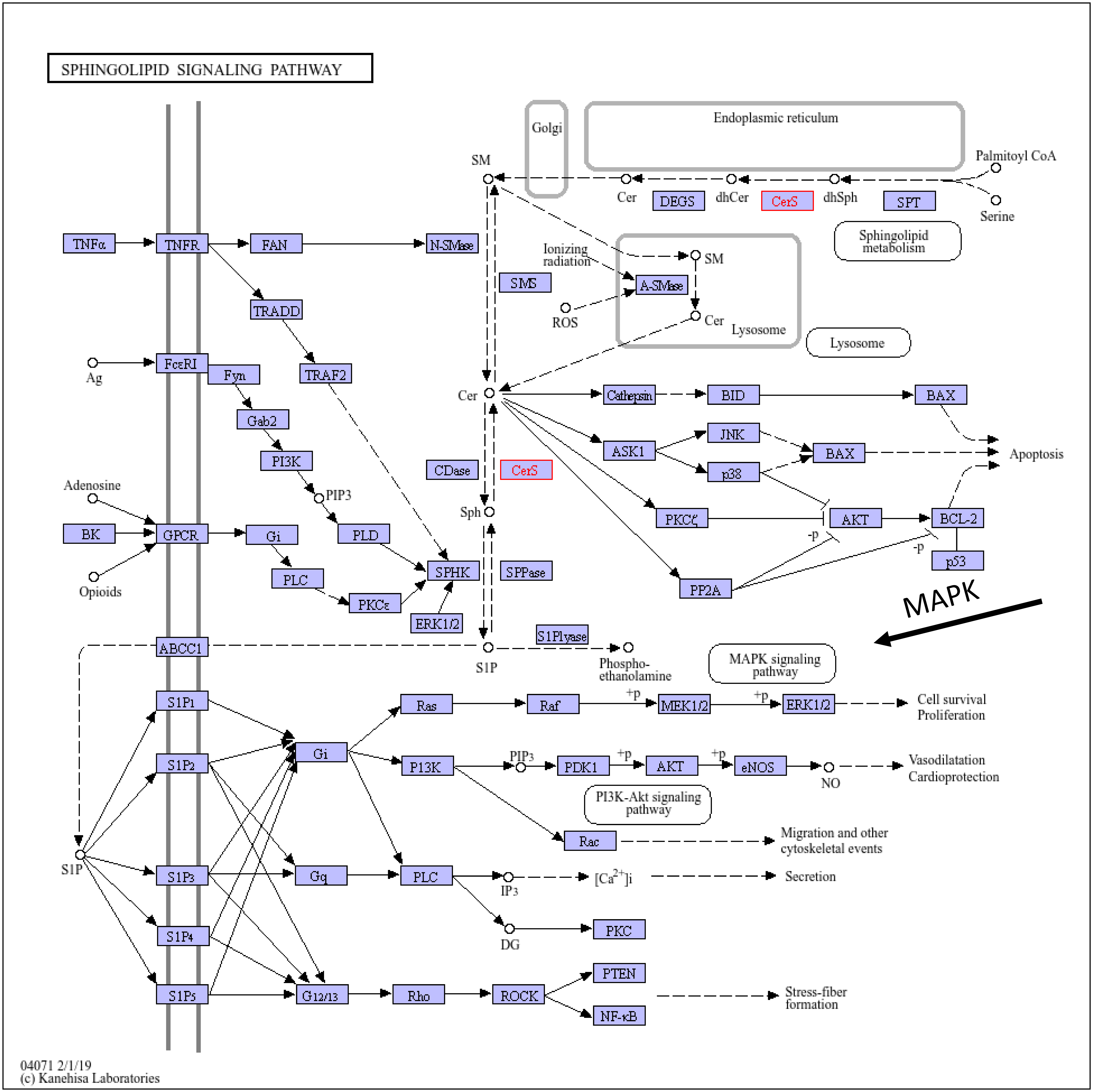
SPHINGOLIPID SIGNALING PATHWAY
Golgi
Endoplasmic reticulum
Palmitoyl CoA
SM
Cer
DEGS
dhCer
CerS
dhSph
SPT
Serine
Sphingolipid metabolism
TNFα
TNFR
FAN
N-SMase
Ionizing radiation
SM
SMS
A-SMase
ROS
Cer
Lysosome
Lysosome
TRADD
Ag
FcεRI
Fyn
TRAF2
Cer
Cathepsin
BID
BAX
Gab2
JNK
ASK1
BAX
Apoptosis
PI3K
p38
CDase
CerS
PIP3
Adenosine
Sph
PKCζ
AKT
BCL-2
BK
GPCR
Gi
PLD
-p
-p
Opioids
PLC
SPHK
SPPase
p53
PKCε
PP2A
ERK1/2
MAPK
S1Plyase
ABCC1
S1P
Phospho-ethanolamine
MAPK signaling pathway
S1P1
Ras
Raf
+p
MEK1/2
+p
ERK1/2
Cell survival Proliferation
Gi
S1P2
PI3K
PIP3
PDK1
+p
AKT
+p
eNOS
NO
Vasodilatation Cardioprotection
PI3K-Akt signaling pathway
Rac
Migration and other cytoskeletal events
S1P
S1P3
Gq
PLC
IP3
[Ca2+]i
Secretion
DG
PKC
S1P4
PTEN
S1P5
G12/13
Rho
ROCK
Stress-fiber formation
NF-κB
04071 2/1/19
(c) Kanehisa Laboratories

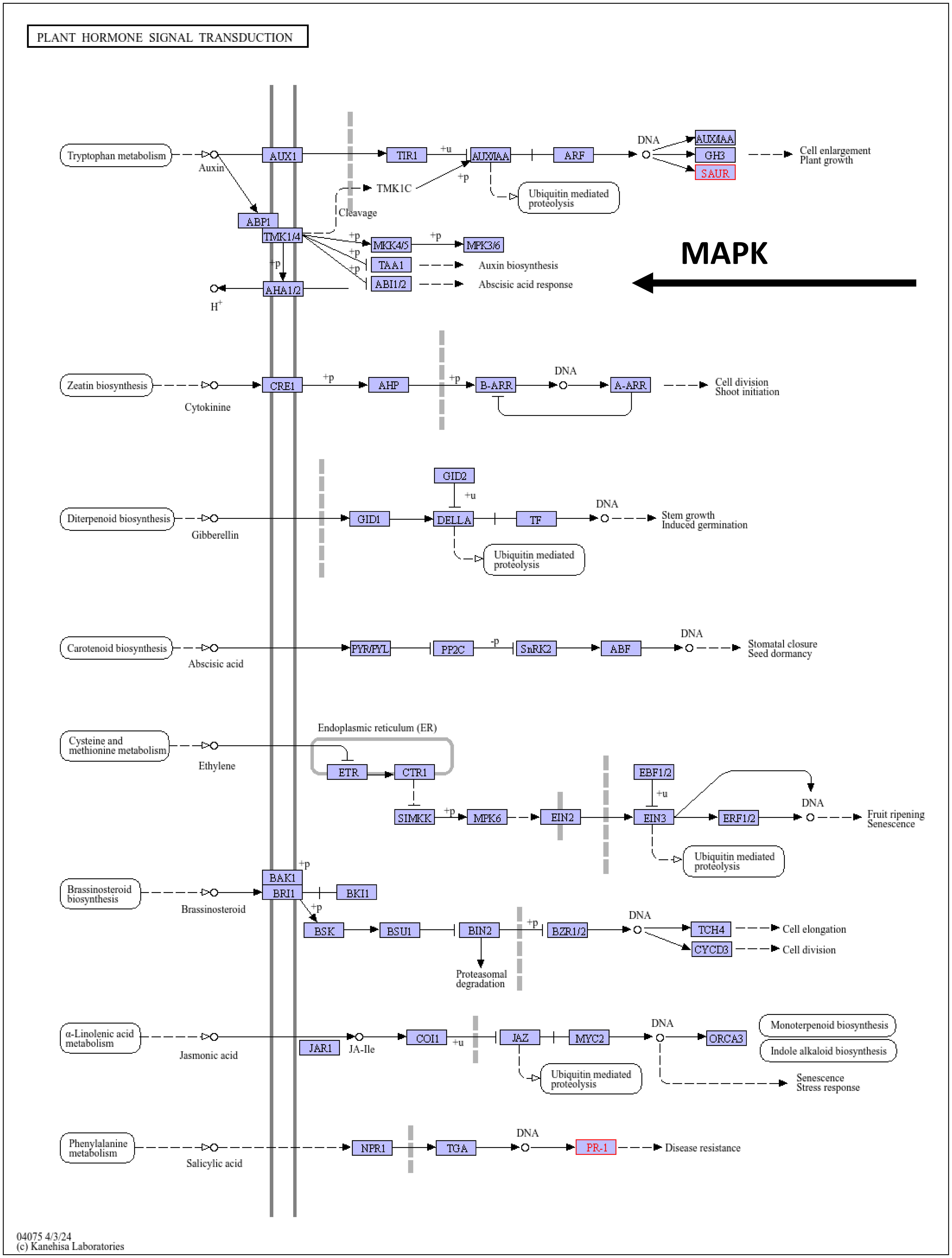

Figure S6: - The KEGG sphingolipid singling pathway and plant hormonal signaling pathway which included MAPK.
